# Enhanced Oxidative Phosphorylation Driven by TACO1 Mitochondrial Translocation Promotes Stemness and Cisplatin Resistance in Bladder Cancer

**DOI:** 10.1002/advs.202408599

**Published:** 2024-12-10

**Authors:** Minhua Deng, Zhaohui Zhou, Jiawei Chen, Xiangdong Li, Zefu Liu, Jingwei Ye, Wensu Wei, Ning Wang, Yulu Peng, Xin Luo, Lijuan Jiang, Fangjian Zhou, Xianchong Zheng, Zhuowei Liu

**Affiliations:** ^1^ State Key Laboratory of Oncology in South China Guangdong Provincial Clinical Research Center for Cancer Sun Yat‐sen University Cancer Center Guangzhou 510060 China; ^2^ Department of Urology Sun Yat‐Sen University Cancer Center Guangzhou 510060 China; ^3^ Department of Urology Shunde Hospital Southern Medical University (The First People's Hospital of Shunde Foshan) Foshan 528000 China; ^4^ Department of Urology Sun Yat‐sen University Cancer Center Gansu Hospital Lanzhou 730050 China

**Keywords:** bladder cancer, cancer stemness, chemoresistance, oxidative phosphorylation, TACO1

## Abstract

Chemoresistance poses a critical obstacle in bladder cancer (BCa) treatment, and effective interventions are currently limited. Elevated oxidative phosphorylation (OXPHOS) has been linked to cancer stemness, a determinant of chemoresistance. However, the mechanisms underlying increased OXPHOS during cancer cell chemoresistance remain unclear. This study revealed that the mitochondrial translational activator of cytochrome oxidase subunit 1 (TACO1) is linked to stemness and cisplatin resistance in BCa cells. Mechanistically, mitochondrial TACO1 enhances the translation of the mitochondrial cytochrome c oxidase I (MTCO1), promoting mitochondrial reactive oxygen species (mtROS) by upregulating OXPHOS, consequently driving cancer stemness and cisplatin resistance. Intriguingly, the mitochondrial translocation of TACO1 is mediated by the heat shock protein 90 β (HSP90β), a process that requires circFOXK2 as a scaffold for the TACO1‐HSP90β interaction. The mutations at the binding sites of TACO1‐circFOXK2‐HSP90β disturb the ternary complex and inhibit cancer stemness and cisplatin resistance in BCa cells by suppressing the MTCO1/OXPHOS/mtROS axis. Clinically, BCa patients with increased mitochondrial TACO1 expression respond poorly to cisplatin treatment. This study elucidates the mechanisms by which TACO1 promotes BCa stemness and cisplatin resistance, providing a potential target for mitigating cisplatin resistance for BCa and a biomarker for predicting cisplatin response.

## Graphical abstract

Chemoresistance is a significant cause of death in patients with advanced bladder cancer (BCa), but its underlying mechanisms remain unclear. This study links TACO1 to cisplatin resistance in BCa patients, exploring the role of the HSP90β‐circFOXK2‐TACO1 complex in cisplatin resistance and the potential of mitochondrial TACO1 as a new therapeutic target for overcoming cisplatin resistance in BCa patients.

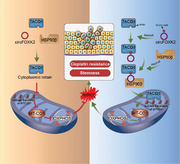

## 1 Introduction

Bladder cancer (BCa) is one of the most common malignancies within the urinary system, with muscle‐invasive bladder cancer (MIBC) being a particularly aggressive subtype.^[^ ^1^ ^]^ Following radical cystectomy, MIBC patients frequently encounter lymph node metastasis and, in some cases, distant organ metastasis, leading to a 50% mortality rate.^[^ ^2^ ^]^ Despite ongoing advancements in treatment, cisplatin‐based chemotherapy remains the primary therapeutic option for advanced and metastatic BCa. Unfortunately, BCa frequently develops resistance to cisplatin, resulting in cancer recurrence and a reduced 5‐year overall survival (OS) rate of less than 40%.^[^ ^2^, ^3^ ^]^ Therefore, overcoming cisplatin resistance in BCa presents a formidable clinical challenge.

During tumorigenesis, a distinct subgroup of cancer cells possessing stem cell‐like properties, referred to as cancer stem cells (CSCs), emerges. CSCs are often characterized by the expression of specific surface markers such as CD44, CD133, or acetaldehyde dehydrogenase (ALDH).^[^ ^4^ ^]^ CSCs play a crucial role in developing resistance to chemotherapy within cancer. Although chemotherapy is effective in eliminating the majority of cancer cells in some cases, CSCs often evade eradication, which is considered critical for cancer recurrence and progression, particularly following chemotherapy in cancers such as BCa.^[^ ^5^ ^]^ However, the mechanisms by which cancer cells maintain their stem cell‐like properties during the development of chemotherapy resistance are not yet fully understood.

Energy metabolism reprogramming often occurs during cancer progression, enabling cancer cells to adapt to the complex and dynamic tumor microenvironment and external stimuli. Notably, the active metabolic reshaping process in CSCs provides them with sufficient energy to maintain their traits. Recent studies have emphasized that oxidative phosphorylation (OXPHOS) hyperactivity is crucial for preserving the stem cell‐like traits of cancer cells and their resistance to chemotherapy, as observed in both solid tumors^[^ ^6^ ^]^ and hematological malignancies.^[^ ^7^ ^]^ Eliminating the excessive level of OXPHOS in cancer cells has become one of the potential therapeutic approaches to overcome chemoresistance in pancreatic cancer.^[^ ^8^ ^]^ OXPHOS primarily occurs in the mitochondria and heavily relies on the mitochondrial respiratory chain, which comprises five protein complexes (complexes I–V).^[^ ^9^ ^]^ The assembly of these protein complexes in mammals is an exceedingly complex process, involving the intricate regulation of over 130 proteins.^[^ ^10^ ^]^ Reduced/elevated expression of these assembly factors is associated with a decrease/increase in OXPHOS.^[^ ^11^ ^]^ Meanwhile, elevated expression of these factors can lead to cancer progression.^[^ ^11^, ^12^ ^]^ Despite these findings, the mechanisms underlying the upregulation of OXPHOS during cancer cell chemoresistance remain unclear.

In this study, we examined the expression of complex I–V assembly factors in BCa and identified a correlation between elevated mitochondrial translational activator of cytochrome oxidase subunit 1 (TACO1) expression and BCa progression, particularly concerning BCa cell stemness and cisplatin resistance. Additionally, we delineated the mechanism by which TACO1 enhances stemness and cisplatin resistance through the mitochondrial cytochrome c oxidase I (MTCO1)/OXPHOS/mitochondrial reactive oxygen species (mtROS) axis. Crucially, this process hinges on the mitochondrial translocation of TACO1, which is facilitated by the TACO1‐circFOXK2‐the heat shock protein 90 β (HSP90β) ternary complex, where circFOXK2 serves as a scaffold. Furthermore, the expression of mitochondrial TACO1 is inversely correlated with the response of BCa patients to cisplatin treatment.

## 2 Results

### 2.1 OXPHOS Enhances Stemness and Cisplatin Resistance in BCa Cells

To investigate the correlation between OXPHOS and the BCa cisplatin resistance, we initially established cisplatin‐resistant (CIS) cell lines of T24 and UMUC3, named T24‐CIS and UMUC3‐CIS, respectively (Figure S1A, Supporting Information). Similar to previous studies, the resistance index of these resistant cells is approximately four, indicating the successful establishment of the cisplatin‐resistant cells.^[^ ^13^ ^]^ The oxygen consumption rate (OCR) exhibited by T24‐CIS and UMUC3‐CIS was higher than that of their respective parental cell lines (Figure S1B, Supporting Information), suggesting a correlation between elevated OXPHOS and the development of cisplatin resistance in BCa cells. It is noteworthy that the administration of oligomycin, a specific inhibitor that suppresses cellular OXPHOS by targeting the F0/F1 ATPase complex,^[^ ^14^ ^]^ impaired both the stemness and cisplatin resistance in T24 and UMUC3 cells (Figure S1C–F, Supporting Information), as well as their corresponding CIS cell lines (Figure S1G–J, Supporting Information). These results suggested that elevated OXPHOS enhances the stem cell phenotype and cisplatin resistance in BCa cells.

### 2.2 Increased Expression of TACO1 is Associated with BCa Progression

**Figure 1:**
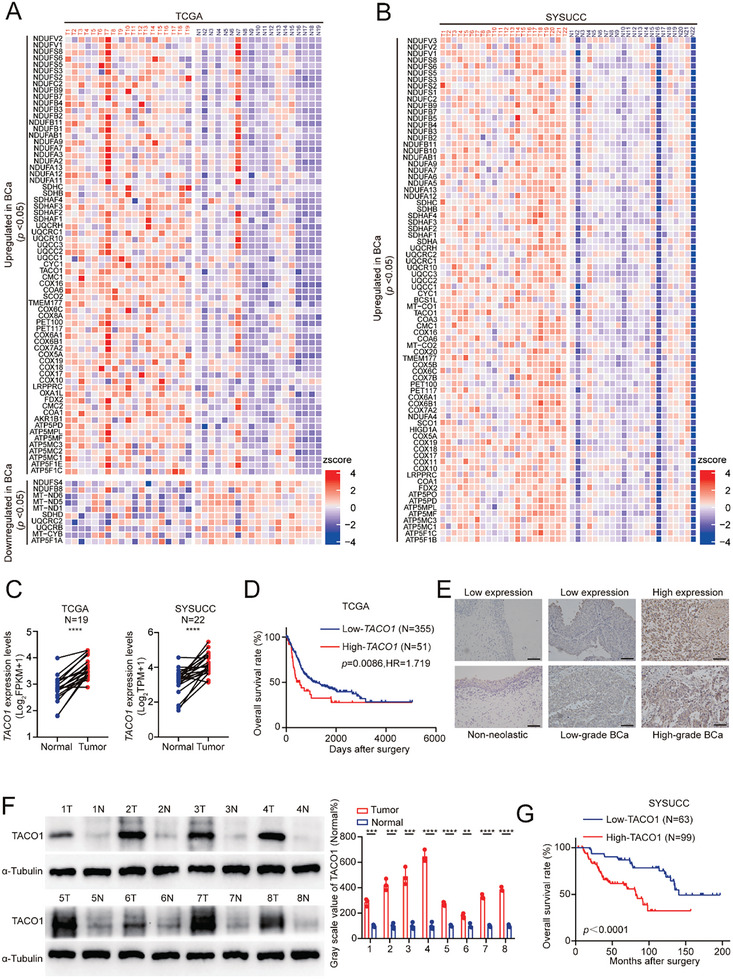
Elevated expression of TACO1, a complex IV assembly factor, is associated with BCa progression. A,B) The expression profiles of complex I‐V assembly factors in BCa tissues from TCGA and SYSUCC (paired sample *t*‐test). C) *TACO1* expression in BCa tissues compared with matched normal tissues from TCGA and SYSUCC (****p < 0.0001, paired sample *t*‐test). D) The correlation between TACO1 expression and the prognosis of BCa patients as analyzed within the TCGA database. E) Representative images of immunohistochemistry (IHC) staining for TACO1 in normal bladder tissue, low‐grade BCa, and high‐grade BCa. Scale bar, 100 µm. F) (Left) Western blot analysis was used to detect TACO1 expression in fresh BCa tissues and their paired normal tissues; (Right) Quantification of grayscale values for the specified proteins was conducted. Data are shown as mean ± SD, n = 3 (**p < 0.01, ***p < 0.001, ****p < 0.0001, independent Student's *t*‐test). G) Kaplan‐Meier curves demonstrated the correlation between TACO1 expression and the survival of BCa patients from SYSUCC.

Given that the completion of the OXPHOS process relies on mitochondrial respiratory chain complex proteins,^[^ ^9^ ^]^ we speculate that the expression of these assembly factors is closely associated with the levels of OXPHOS. Subsequently, we analyzed the expression levels of complex I‐V assembly factors in BCa tissue sequencing data obtained from the Cancer Genome Atlas (TCGA) and Sun Yat‐sen University Cancer Center (SYSUCC)^[^ ^15^ ^]^ (**Figure** **1**A,B; Figure S2A,B, Supporting Information). Notably, most complex protein assembly factors, specifically *SDHAF1, SDHAF3, UQCC3, TACO1*, *COA6*, and *TMEM177*, were upregulated in BCa tissues (fold change > 1.2, *p* < 0.05, Figure 1C; Figure S2C, Supporting Information). Among these six factors, elevated expression of *TACO1* was found to be significantly correlated with an unfavorable prognosis in patients with BCa (Figure 1D; Figure S2D, Supporting Information). Compared with normal bladder tissues and low‐grade BCa tissues, the protein levels of TACO1 are significantly elevated in high‐grade BCa tissues (Figure 1E,F). Furthermore, the expression levels of TACO1 are upregulated in most BCa cell lines and BCa‐CIS cells (Figure S2E,F, Supporting Information). High TACO1 protein levels were significantly associated with a poorer prognosis in BCa patients (Figure 1G). Additionally, elevated TACO1 protein levels are associated with higher histological grade, T stage, lymph node metastasis, and a higher likelihood of cancer recurrence in BCa (Table S1, Supporting Information). Multivariate regression analysis indicated that elevated TACO1 protein is an independent prognostic factor for BCa patients (Table S2, Supporting Information). Taken together, these results suggest that the upregulation of TACO1 is correlated with the progression of BCa.

### 2.3 TACO1 Promotes Stemness and Cisplatin Resistance in BCa Cells Through the MTCO1/OXPHOS Axis

**Figure 2:**
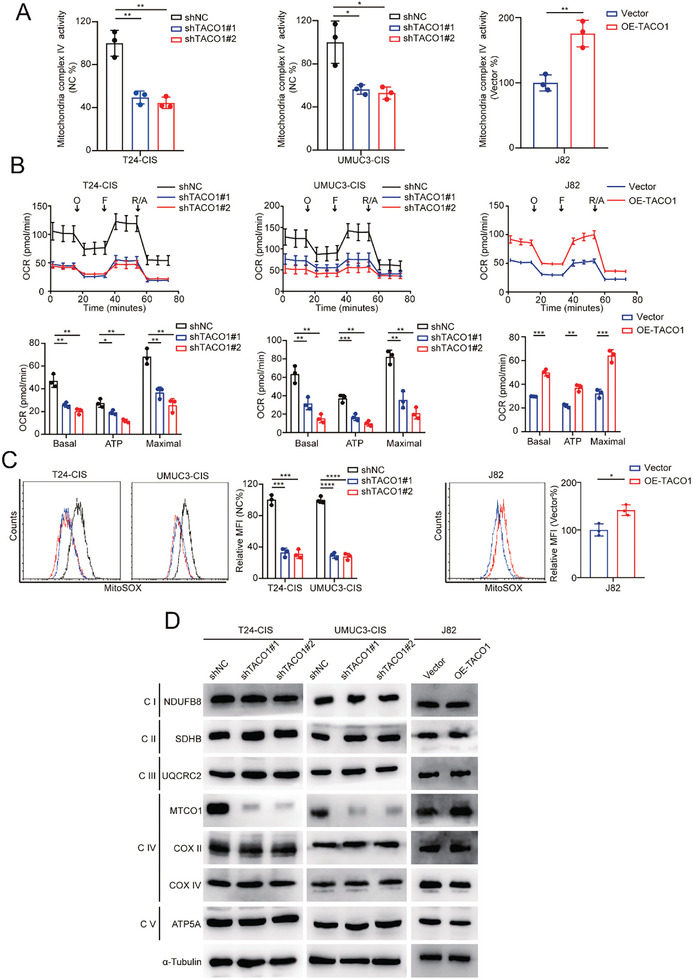
TACO1 increases OXPHOS and MTCO1 expression levels in BCa cells. A) The relationship between the expression level of TACO1 and the activity of mitochondrial complex IV in BCa cells. B) Seahorse assay was used to detect the impact of knocking down or overexpressing TACO1 on oxygen consumption rate (OCR) in the corresponding cells. O: Oligomycin; F: FCCP; R/A: Rotenone & Antimycin A. Data are shown as mean ± SD, n = 3 (**p < 0.01, ***p < 0.001, independent Student's *t*‐test). C) Flow cytometry was used to assess the levels of MitoSOX in each treatment group. Data are shown as mean ± SD, n = 3 (*p < 0.05, ***p < 0.001, ****p < 0.0001, independent Student's *t*‐test). D) Western blot analysis showed alterations in indicated mitochondrial complex proteins following knockdown or overexpression of TACO1 in T24‐CIS, UMUC3‐CIS, or wild‐type J82 cells.

Next, we investigated whether the association between elevated TACO1 expression and BCa malignancy is due to its involvement in regulating OXPHOS, given its role in complex IV assembly. To this end, we established TACO1 stable knockdown cells in T24‐CIS and UMUC3‐CIS cells and TACO1 stable overexpression cells in wild‐type J82 cells (Figure S3A). TACO1 knockdown decreased the mitochondria complex IV activity and OCR of T24‐CIS and UMUC3‐CIS cells, while TACO1 overexpression increased the mitochondria complex IV activity and OCR of wild‐type J82 cells, but had no notable impact on extracellular acidification rate (ECAR; **Figure** **2**A,B; Figure S3B, Supporting Information). As a by‐product of the OXPHOS process,^[^ ^6a^ ^]^ mtROS levels are decreased in BCa‐CIS cells after knockdown of TACO1 (Figure 2C). Conversely, in J82 cells, overexpression of TACO1 resulted in an upregulation of mtROS (Figure 2C). Previous research reported that TACO1 decrease causes a significant reduction in the expression of MTCO1 in mice.^[^ ^16^ ^]^ Similarly, we have found that TACO1 knockdown inhibited MTCO1 expression in BCa‐CIS cells, while TACO1 overexpression enhanced the expression of MTCO1 in wild‐type J82 cells (Figure 2D; Figure S3C, Supporting Information), which is a crucial component of complex IV. Notably, the expression of other essential factors in the mitochondrial respiratory chain was not significantly affected by TACO1 knockdown or overexpression (Figure 2D). Given that TACO1 had no significant effect on MTCO1 mRNA levels (Figure S3D, Supporting Information), we hypothesized that TACO1 might influence MTCO1 translation. Indeed, polysome profiling analysis revealed that TACO1 knockdown decreased the mRNA levels of MTCO1 within polysomes (Figure S3E,F, Supporting Information), affirming the role of TACO1 in enhancing the translational efficiency of MTCO1. These findings suggest that TACO1 promotes MTCO1 protein expression, enhancing the mitochondrial complex IV activity, OXPHOS, and mtROS.

**Figure 3:**
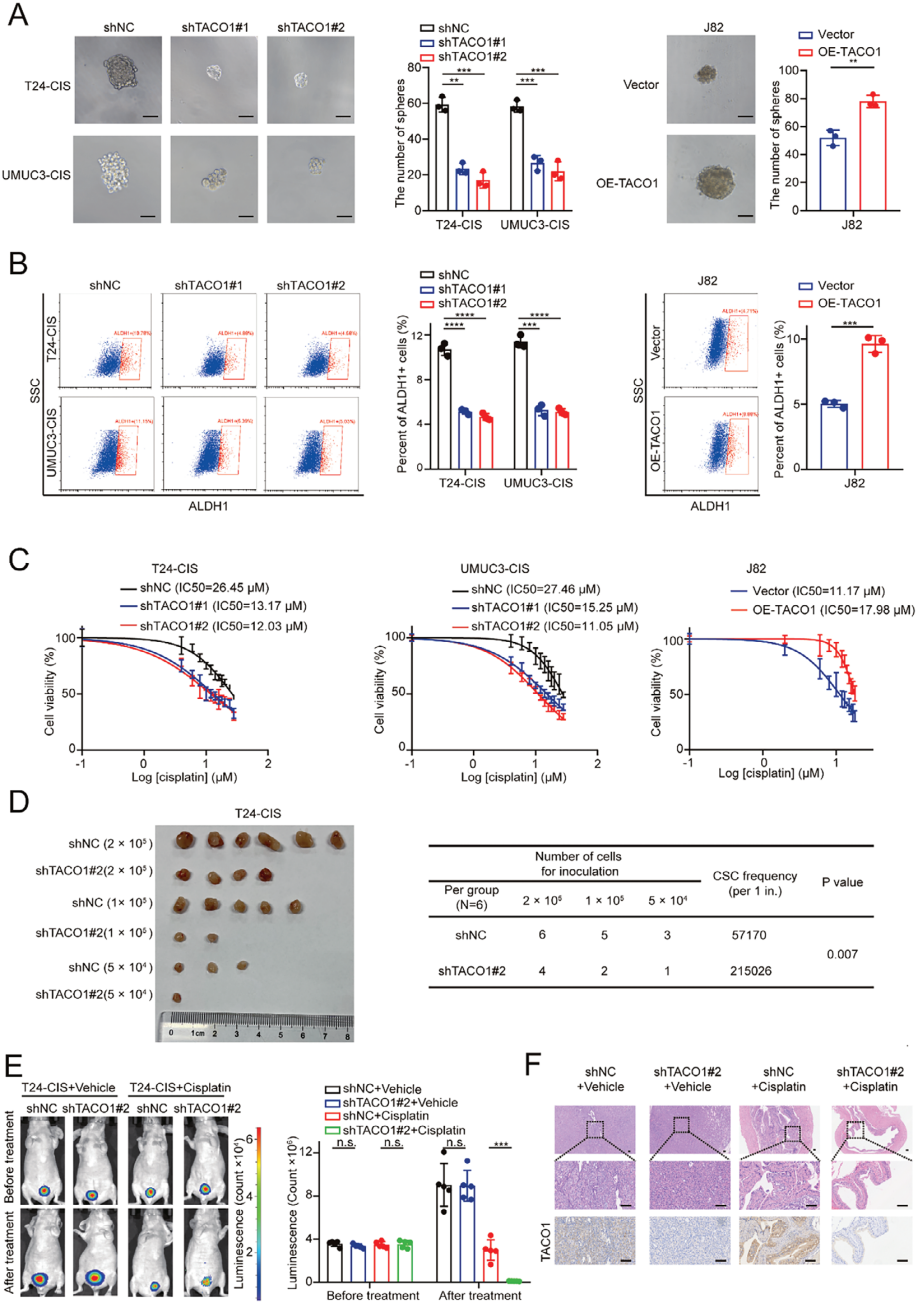
TACO1 promotes stemness and cisplatin resistance in BCa cells. A) Effect of TACO1 on the sphere‐forming capacity of BCa cells. Scale bar, 100 µm. Data are shown as mean ± SD, n = 3 (***p < 0.001, ****p < 0.0001, independent Student's *t*‐test). B) Flow cytometry assay showed the proportion of ALDH1+ cells among BCa cells. Data are shown as mean ± SD, n = 3 (*p < 0.05, ***p < 0.001, independent Student's *t*‐test). C) The resistance of corresponding BCa cells to cisplatin was assessed. Cellular viability was determined using the cell counting kit 8 (CCK‐8) assay, and the half‐maximal inhibitory concentration (IC50) values were calculated through nonlinear regression analysis. Data are shown as mean ± SD, n = 3. D) Tumor initiation assays were conducted using T24‐CIS‐shNC and T24‐CIS‐shTACO1 cells (Left panel). Limiting dilution assays were performed to determine the frequency of tumorigenic cells (Right panel). E,F) Representative bioluminescence images (Left), HE staining, and IHC images (Right), along with statistical results of the bioluminescence signals (Middle) for the bladder orthotopic xenograft model, were presented for subgroups treated with either vehicle or cisplatin. Scale bar, 100 µm. Data are shown as mean ± SD, n = 5 (***p < 0.001, n.s. = non‐significant, independent Student's *t*‐test).

Given that OXPHOS enhances stemness and cisplatin resistance in BCa cells (Figure S1B–F, Supporting Information), we hypothesized that TACO1 may also promote these traits. As anticipated, TACO1 knockdown inhibited both stemness and cisplatin resistance in BCa‐CIS cells, while TACO1 overexpression had the opposite effect in wild‐type J82 cells (**Figure** **3**A–C; Figure S4A, Supporting Information). However, TACO1 had no significant effect on the proliferation ability of BCa cells (Figure S4B,C, Supporting Information). Moreover, a notable downregulation of SOX2, a key driver gene of cancer stemness,^[^ ^17^ ^]^ was observed following the knockdown of TACO1 in BCa‐CIS cells. In contrast, overexpression of TACO1 in wild‐type J82 cells promotes the expression of SOX2 (Figure S4D, Supporting Information). Consistent with our in vitro findings, data from subcutaneous tumor formation and orthotopic BCa models demonstrated that TACO1 knockdown inhibited the tumor‐initiating capacity and cisplatin resistance of T24‐CIS cells in vivo (Figure 3D–F).

To confirm that TACO1's role in promoting stemness and cisplatin resistance is mediated through the MTCO1/OXPHOS/mtROS axis, we transfected MTCO1 plasmids into TACO1 knockdown T24‐CIS cells and treated them with GYY4137 (a complex IV inhibitor^[^ ^18^ ^]^) or MitoTEMPO (a mitochondria‐targeted antioxidant) (Figure S5A, Supporting Information). The decrease in complex IV activity, OCR, mtROS, percent of ALDH1+ and CD44+, expression levels of SOX2, and cisplatin resistance caused by TACO1 knockdown was substantially restored upon reintroducing MTCO1 expression, but this effect was compromised in the presence of GYY4137 (Figure S5B–I, Supporting Information). Interestingly, eliminating mtROS without affecting complex IV activity and OXPHOS can also inhibit the restorative effects of MTCO1 overexpression (Figure S5B–I, Supporting Information). Furthermore, eliminating mtROS can also inhibit the cisplatin resistance of T24‐CIS cells (Figure S5J, Supporting Information). These results collectively indicate that TACO1 promotes the expression of the MTCO1 protein, enhancing complex IV activity and OXPHOS. This, in turn, upregulates mtROS, subsequently promoting stemness and cisplatin resistance in BCa cells.

### 2.4 MTCO1 Protein Expression Mediated by TACO1 Mitochondrial Translocation Requires HSP90β

**Figure 4:**
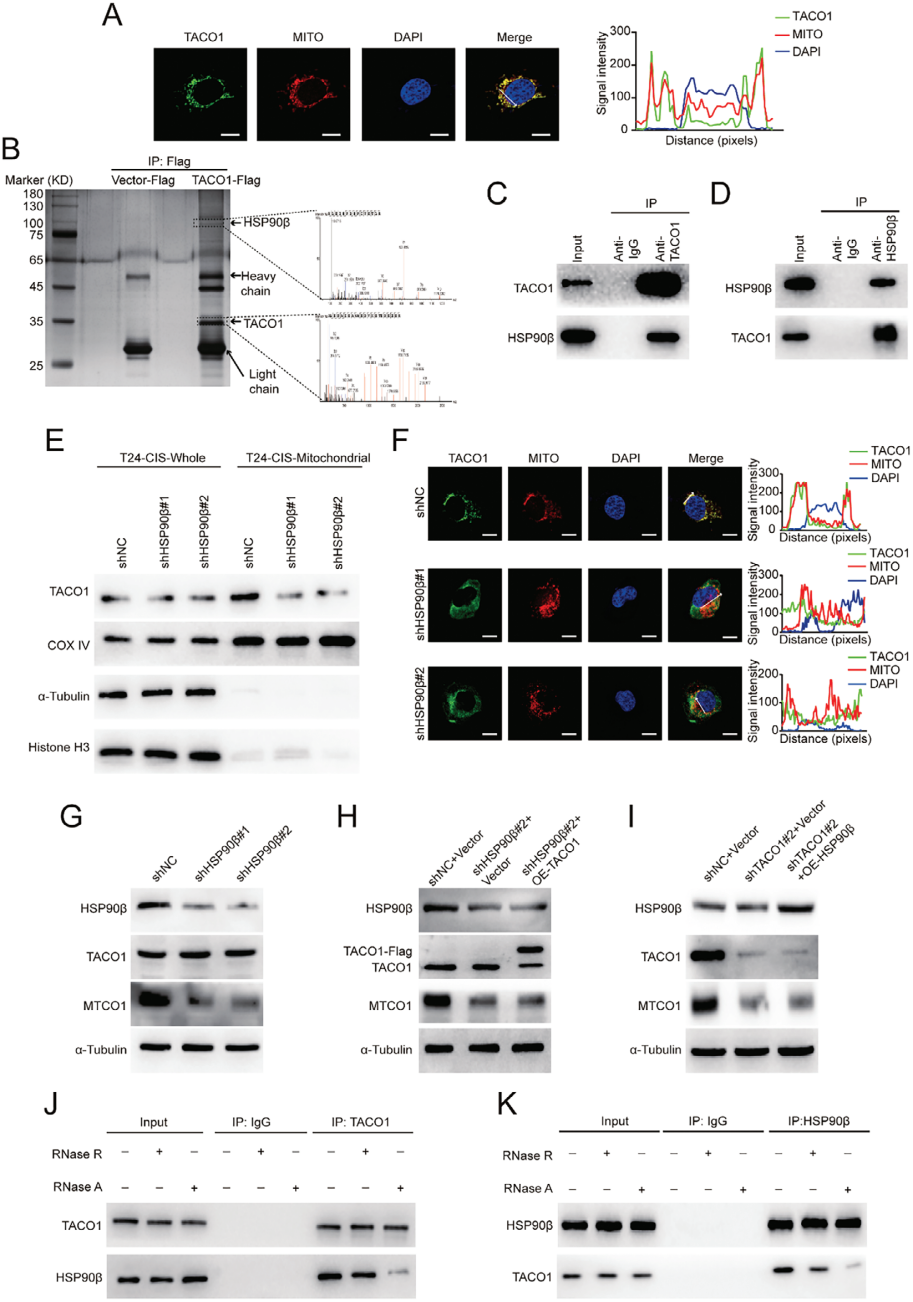
TACO1 translocates to the mitochondria and influences MTCO1 expression by binding to HSP90β. A) IF was performed using anti‐TACO1 (green) and MitoTracker (red). Nuclei were stained using DAPI. Line graphs indicate the signal intensity of each protein along the arrow bars. Scale bar, 10 µm. B) Silver staining revealed TACO1‐binding proteins in T24‐CIS cells (Left), and mass spectrometry identified the peptide sequence of HSP90β (Right). C,D) co‐IP was performed to confirm the interaction between TACO1 and HSP90β in T24‐CIS cells. E) Western blot showed the effect of knockdown of HSP90β on TACO1 expression. COX IV was used as a mitochondrial marker, α‐Tubulin was used as a cytoplasm marker and Histone‐H3 was used as a nuclear marker. F) IF showed the effect of knockdown of HSP90β on the co‐localization of TACO1 and MitoTracker. Line graphs indicate the signal intensity of each protein along the arrow bars. Scale bar, 10 µm. G–I) Western blot showed the alterations in the expression of TACO1, HSP90β, and MTCO1 in indicated subgroups. J,K) co‐IP experiments confirmed the binding between TACO1 and HSP90β post RNase R or RNase A treatment.

While TACO1 predominantly localizes within the mitochondria of T24‐CIS cells (**Figure** **4**A), similar to its localization in HEK293 cells,^[^ ^19^ ^]^ the mechanism by which this nuclear‐encoded protein enters the mitochondria remains unclear. To address this, we employed coimmunoprecipitation (co‐IP) and mass spectrometry (MS) to identify proteins that interact with TACO1 in T24‐CIS cells. In the TACO1 interactome, our focus was on HSP90β (Figure 4B, **PeptideAtlas ID: PASS04824)**, given the established role of HSP90β in facilitating the mitochondrial import of cytosolic proteins.^[^ ^20^ ^]^ Notably, HSP90β was significantly upregulated in BCa tissues from TCGA and SYSUCC (Figure S6A,B, Supporting Information). Furthermore, elevated HSP90β expression was associated with a poor prognosis in BCa patients (Figure S6C, Supporting Information). The interaction of endogenous TACO1 and HSP90β was confirmed by co‐IP (Figure 4C,D). Remarkably, the knockdown of HSP90β did not impact the overall levels of TACO1 protein but led to a decrease in the mitochondrial levels of TACO1 protein (Figure 4E). In line with this, the knockdown of HSP90β disrupted the mitochondrial localization of TACO1 protein, as evidenced by immunofluorescence (IF) analysis (Figure 4F). It's worth noting that HSP90β knockdown reduced the expression of MTCO1 protein (Figure 4G), and this effect could not be reversed by overexpressing TACO1 (Figure 4H). Similarly, the decrease in MTCO1 protein caused by TACO1 knockdown could not be restored by overexpressing HSP90β (Figure 4I). These data indicate that HSP90β facilitates the mitochondrial translocation of TACO1, which in turn promotes the expression of MTCO1 protein.

### 2.5 CircFOXK2 Promotes the MTCO1/OXPHOS Axis by Acting as a Scaffold for the TACO1‐HSP90β Interaction

**Figure 5:**
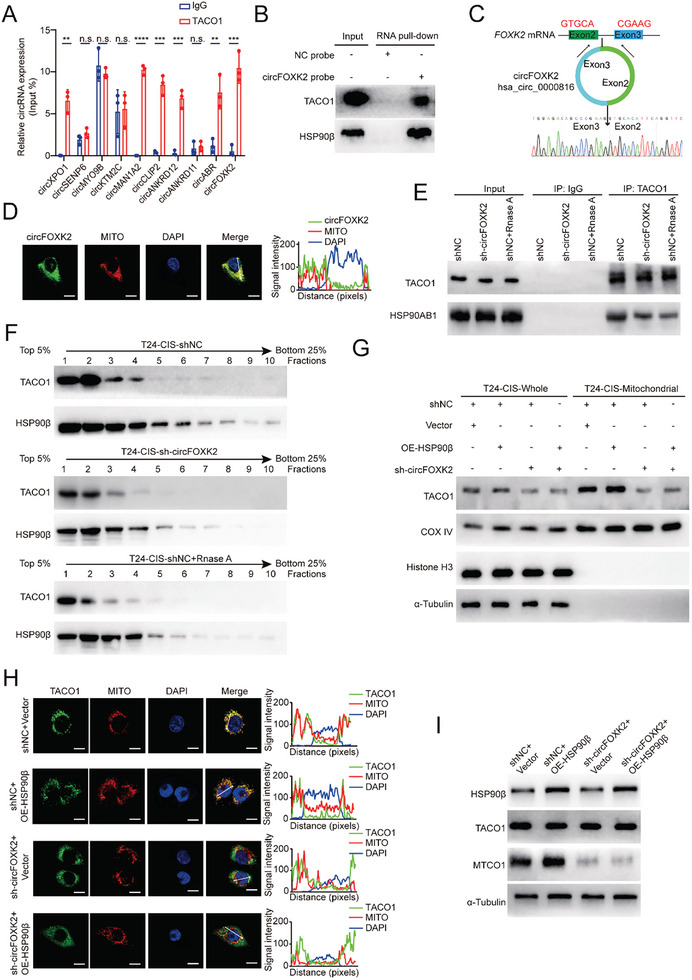
circFOXK2 acts as a molecular scaffold connecting TACO1 and HSP90β, thereby facilitating the mitochondrial translocation of TACO1 and MTCO1 expression. A) RIP‐qPCR analysis was used to reveal the binding between TACO1 and circRNAs in T24‐CIS cells. Data are shown as mean ± SD, n = 3 (**p < 0.01, ***p < 0.001, ****p < 0.0001, n.s. = non‐significant, independent Student's *t*‐test). B) RNA pull‐down showed that circFOXK2 could combine with both TACO1 and HSP90β in T24‐CIS cells. C) Sanger sequencing validated the back splicing junction site of circFOXK2 in T24‐CIS cells. D) IF‐RNA fluorescence in situ hybridization (FISH) validation of circFOXK2 and MitoTracker co‐localization in T24‐CIS cells. Nuclei were stained using DAPI. Line graphs indicate the signal intensity of each protein along the arrow bars. Scale bar, 10 µm. E) co‐IP revealed the interaction between TACO1 and HSP90β following circFOXK2 knockdown or RNase A treatment in T24‐CIS cells. F) Glycerol gradient sedimentation and western blot showed the distribution of TACO1 and HSP90β in a 5–25% gradient of glycerol after the indicated treatment in T24‐CIS cells. G) Western blot showed the distribution of TACO1 expression in each subgroup. COX IV was used as a mitochondrial marker, α‐Tubulin was used as a cytoplasm marker and Histone‐H3 was used as a nuclear marker. H) IF showed the co‐localization of TACO1 and MitoTracker in each subgroup. Line graphs indicate the signal intensity of each protein along the arrow bars. Scale bar, 10 µm. I) Western blot displayed the impact of circFOXK2 and HSP90β on the expression of MTCO1 in T24‐CIS cells.

To further explore the TACO1‐HSP90β interaction, we conducted GST pull‐down. However, TACO1 did not directly bind to HSP90β (Figure S7A). Considering that TACO1 is an RNA‐binding protein, we hypothesized that the TACO1‐HSP90β interaction might require the involvement of specific RNAs. For elucidation, we performed co‐IP with T24‐CIS cells treated with RNase R (which mainly degrades linear RNA) and RNase A (which degrades total RNA), separately. Interestingly, the TACO1‐HSP90β interaction was significantly disrupted by RNase A treatment but remained less affected by RNase R treatment (Figure 4J,K). This finding suggested that the interaction between TACO1 and HSP90β may rely on circRNAs. To further identify the circRNAs that specifically bind with TACO1, we performed RNA immunoprecipitation sequencing (RIP‐Seq) in T24‐CIS cells. By analyzing the RIP‐Seq results **(BioProject ID: PRJNA953625)**, we found that 10 circRNAs were obviously enriched in the anti‐TACO1 group compared with the anti‐IgG group (circRNA reads in the anti‐TACO1 group > 10 and Log_2_ Fold change > 1.5). To validate the results of RIP‐Seq, we performed RIP‐qPCR assays and the results showed that only 6 circRNAs were enriched in the anti‐TACO1 group compared with the anti‐IgG group, including circXPO1 (hsa_circ_0001017), circABR (hsa_circ_0007919), circMAN1A2 (hsa_circ_0000117), circFOXK2 (hsa_circ_0000816), circANKRD12 (hsa_circ_0000826) and circCLIP2 (hsa_circ_0002755) (**Figure** **5**A). However, among these circRNAs, only circFOXK2 was found to interact with HSP90β, as identified by RIP (Figure S7B, Supporting Information). Additionally, circFOXK2 can bind to both TACO1 and HSP90β, as shown by RNA pull‐down using the circFOXK2 probe (Figure 5B). These data demonstrated that circFOXK2 binds to both TACO1 and HSP90β, so we selected circFOXK2 for further study.

According to the human reference genome (GRCh37/hg19), circFOXK2 (has_circ_0000816) is generated by back‐splicing of FOXK2 exons 2 and 3, resulting in a junction sequence of “CGAAG‐GTGCA” (Figure 5C). Notably, treatments with RNase R and actinomycin D led to a decrease in the levels and stability of FOXK2 mRNA, but circFOXK2 remained less affected (Figure S7C,D). It was observed that circFOXK2 is predominantly localized in the cytoplasm and colocalizes with mitochondria (Figure 5D; Figure S7E). Therefore, we reasoned that the mitochondrial translocation of TACO1, facilitated by HSP90β, requires circFOXK2 to act as a scaffold for the TACO1‐HSP90β interaction. To validate this hypothesis, we designed shRNAs according to the conjunction sequence of circFOXK2. The shRNAs decreased the circFOXK2 expression level and did not affect the expression of the host gene (Figure S7F, Supporting Information). Indeed, knockdown of circFOXK2 not only diminished the TACO1‐HSP90β interaction (Figure 5E,F; Figure S7G, Supporting Information) but also impeded the mitochondrial translocation of TACO1 (Figure S7H,I). Moreover, the reduction in mitochondrial translocation of TACO1 caused by circFOXK2 knockdown could not be restored by overexpressing HSP90β (Figure 5G,H). Consistently, knockdown of circFOXK2 reduced MTCO1 expression, and this effect could not be rescued by overexpressing HSP90β (Figure S7J; Figure 5I). Notably, circFOXK2 knockdown resulted in decreased complex IV activity, OCR, mtROS, stemness, SOX2 expression, and enhanced sensitivity to cisplatin in T24‐CIS cells (Figure S7K–R, Supporting Information). Consistent with in vitro experimental results, knockdown of circFOXK2 can inhibit MTCO1 expression and enhance the sensitivity of BCa cells to cisplatin treatment in orthotopic BCa models (Figure S7S,T, Supporting Information). These findings collectively suggested that circFOXK2 acts as a molecular scaffold for the TACO1‐HSP90β interaction, facilitating the mitochondrial translocation of TACO1. This, in turn, promotes the MTCO1/OXPHOS axis, stemness, and cisplatin resistance in BCa cells.

### 2.6 The TACO1‐circFOXK2‐HSP90β Ternary Complex Requires Both the R196 Residue in TACO1, the AGGU Motif in circFOXK2 and the Q279 Residue in HSP90β

**Figure 6:**
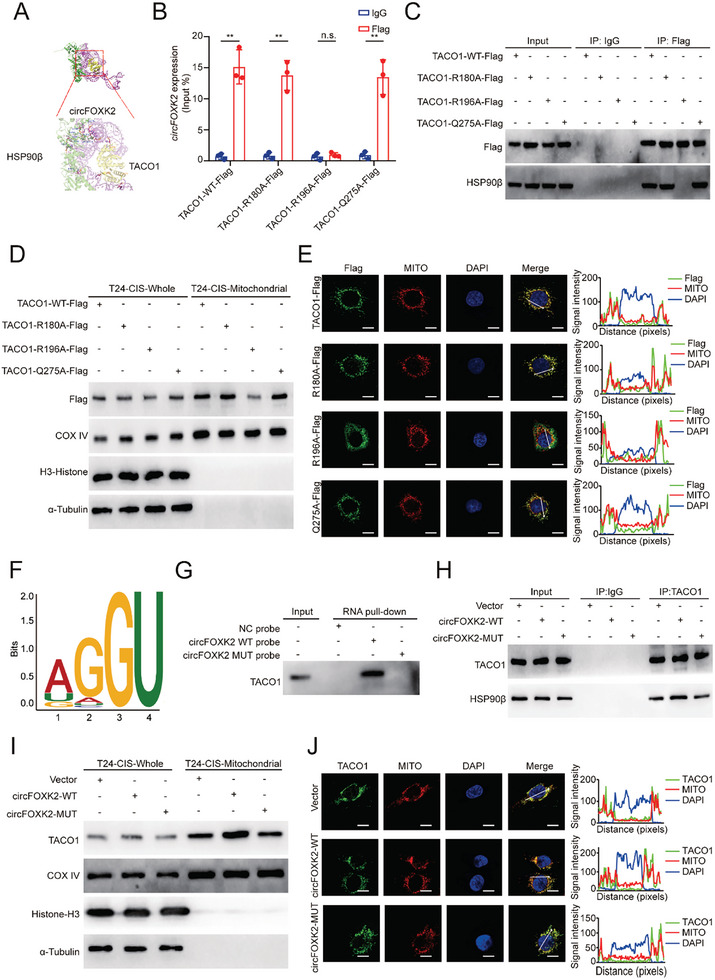
Mutation of the binding site between TACO1 and circFOXK2 disturbs the TACO1/circFOXK2/HSP90β complex and impedes TACO1 mitochondria translocation. A) The binding model among TACO1, circFOXK2 and HSP90β was predicted by molecular docking. B) RIP‐qPCR showed the ability of WT and MUT TACO1 to enrich circFOXK2 in T24‐CIS cells. Data are shown as mean ± SD, n = 3 (**p < 0.01, n.s. = non‐significant, independent Student's *t*‐test). C) co‐IP showed the ability of WT and MUT TACO1 to interact with HSP90β in T24‐CIS cells. D) Western blot displayed the distribution of WT and MUT TACO1 expression levels in mitochondria. COX IV was used as a mitochondrial marker, α‐Tubulin was used as a cytoplasm marker and Histone‐H3 was used as a nuclear marker. E) IF showed the colocalization of WT and MUT TACO1 with MitoTracker in T24‐CIS cells. Nuclei were stained using DAPI. Line graphs indicate the signal intensity of each protein along the arrow bars. Scale bar, 10 µm. F) The AGGU RNA binding motif was specifically enriched by TACO1, as identified by RIP‐Seq. G) RNA pull‐down was performed to assess the binding of WT or MUT circFOXK2 probes to TACO1 in T24‐CIS cells. H) co‐IP showed the effects of WT or MUT circFOXK2 on the binding between TACO1 and HSP90β in T24‐CIS cells. I) Western blot showed the effects of WT or MUT circFOXK2 on TACO1 mitochondrial translocation. J) IF demonstrated the colocalization of TACO1 and MitoTracker in the Vector, circFOXK2‐WT, and circFOXK2‐MUT groups. Line graphs indicate the signal intensity of each protein along the arrow bars. Scale bar, 10 µm.

To further elucidate the role of circFOXK2 as a scaffold for the TACO1‐HSP90β interaction, we conducted molecular docking simulations among the three components. We generated the 3D structure of TACO1 using its amino acid sequences from 63 to 297, as the N‐terminal region (amino acid sequences 1–62) contains a nonsense loop structure (Figure S8A, Supporting Information). The molecular docking model depicts the TACO1‐circFOXK2‐HSP90β ternary complex, wherein the residues R180, R196, and Q275 of TACO1 interact with the nucleotides C330, U174, and G62 of circFOXK2 through three hydrogen bond interactions (**Figure** **6**A; Figure S8B,C and Table S3, Supporting Information). To validate these findings, we generated mutant plasmids for TACO1 based on its residues R180, R196, and Q275, designated as TACO1 (R180A)‐Flag, TACO1 (R196A)‐Flag, and TACO1 (Q275A)‐Flag. Intriguingly, the R196A mutation, but not the R180A or Q275A mutations, in TACO1 reduced its binding to both circFOXK2 and HSP90β (Figure 6B,C; Figure S8D, Supporting Information). Similarly, the R196A mutation, but not the R180A or Q275A mutations, in TACO1 impeded its mitochondrial translocation (Figure 6D,E).

On another note, we reanalyzed the RIP‐Seq data based on TACO1 and found that TACO1 was remarkably enriched at the AGGU motif in the anti‐TACO1 group (Figure 6F). According to the predicted results of molecular docking, the U174 nucleotide on circFOXK2 that binds to the R196 residue of TACO1 is precisely located within the AGGU motif. Therefore, we hypothesized that circFOXK2 facilitates the TACO1‐HSP90β interaction through its AGGU motif (nucleotides 171–174). To investigate this, we designed a circFOXK2 MUT probe for RNA pull‐down experiments (Figure S8E, Supporting Information). The results showed that the circFOXK2‐MUT probe could not interact with TACO1 (Figure 6G). Meanwhile, we generated mutant plasmids for circFOXK2 by altering its AGGU motif, designated as circFOXK2‐MUT (Figure S8F, Supporting Information). As anticipated, overexpression of circFOXK2‐WT increased the interaction between TACO1 and HSP90β and promoted the mitochondrial translocation of TACO1, whereas overexpression of circFOXK2‐MUT did not (Figure 6H–J; Figure S8G, Supporting Information).

To further elucidate the binding between circFOXK2 and HSP90β, we introduced mutations at each of the ten potential binding sites for circFOXK2 on HSP90β, as identified through molecular docking (Table S3, Supporting Information). Subsequent RIP and IP assays revealed that the Q279A mutation, in contrast to other mutations, significantly diminished the binding affinity of HSP90β to both circFOXK2 and TACO1 (Figure S8H,I, Supporting Information).

In summary, these findings suggest that the R196 residue in TACO1, the AGGU motif (nucleotides 171–174) in circFOXK2, and the Q279 residue in HSP90β are crucial for form the TACO1‐circFOXK2‐HSP90β ternary complex, which is pivotal for the mitochondrial translocation of TACO1.

### 2.7 Disrupting the TACO1‐circFOXK2‐HSP90β Ternary Complex Hinders BCa Cell Stemness and Cisplatin Resistance by Blocking the MTCO1/OXPHOS Axis

**Figure 7:**
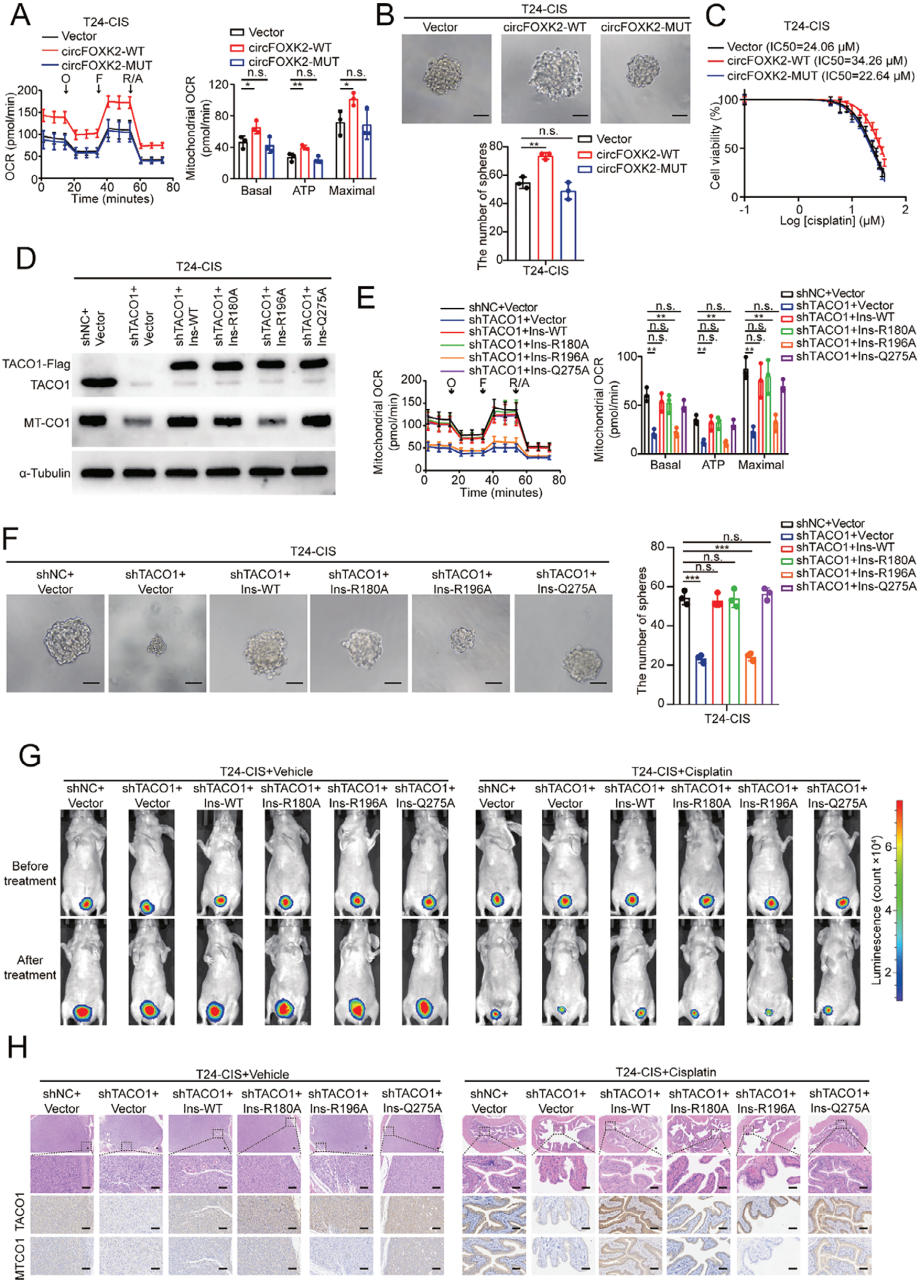
Mutation of the binding site between TACO1 and circFOXK2 reduces the stemness and cisplatin resistance in BCa cells. A) The impact of overexpressing WT or MUT circFOXK2 on the OCR of T24‐CIS cells. Data are shown as mean ± SD, n = 3 (*p < 0.05, **p < 0.01, n.s. = non‐significant, independent Student's *t*‐test). B) Effect of WT or MUT circFOXK2 on the sphere‐forming capacity of T24‐CIS cells. Scale bar, 100 µm. Data are shown as mean ± SD, n = 3 (**p < 0.01, n.s. = non‐significant, independent Student's *t*‐test). C) The impact of overexpressing either WT or MUT circFOXK2 on cisplatin resistance in T24‐CIS cells. Cellular viability was determined by CCK‐8 assay, and nonlinear regression analysis was used to calculate the IC50. Data are shown as mean ± SD, n = 3. D) Western blot demonstrated the impact of MUT TACO1 on the restoration of MTCO1 expression in T24‐CIS‐shTACO1 cells compared to WT TACO1. E) Seahorse analysis showed the impact of MUT TACO1 on the restoration of OCR levels in T24‐CIS‐shTACO1 cells compared to WT TACO1. O: Oligomycin; F: FCCP; R/A: Rotenone & Antimycin A. Data are shown as mean ± SD, n = 3 (**p < 0.01, n.s. = non‐significant, independent Student's *t*‐test). F) The influence of overexpressing WT or MUT TACO1 on the sphere‐forming in T24‐CIS‐shTACO1 cell. Data are shown as mean ± SD, n = 3 (***p < 0.001, n.s. = non‐significant, independent Student's *t*‐test). G,H) The bladder orthotopic xenograft models illustrated the impact of WT and MUT TACO1 on cisplatin resistance in T24‐CIS‐shTACO1 cells. G) Representative bioluminescence images. H) Representative HE staining and IHC images. Scale bar, 100 µm.

Given the crucial role of the TACO1‐circFOXK2‐HSP90β ternary complex in mediating TACO1 mitochondrial translocation, disrupting the formation of this complex is likely to reduce cisplatin resistance in BCa cells. In the orthotopic BCa models, overexpression of HSP90β via shRNA‐insensitive plasmids fully restored the MTCO1 expression levels and cisplatin resistance in T24‐CIS cells, which had been reduced by HSP90β knockdown (Figure S9A,B, Supporting Information). Conversely, the Q279A mutant failed to completely rescue these alterations (Figure S9A,B, Supporting Information). This suggests that the capacity of HSP90β to enhance cisplatin resistance in BCa cells is partially reliant on the Q279 residue interaction with circFOXK2 and TACO1. Similarly, overexpression of circFOXK2‐WT could increase MTCO1 expression, complex IV activity, OCR, mtROS, cancer stemness, cisplatin resistance, and SOX2 expression levels in T24‐CIS cells, but circFOXK2‐MUT could not (**Figure** **7**A–C; Figure S9C–H, Supporting Information). Overexpression of circFOXK2‐WT, but not circFOXK2‐MUT, enhances cisplatin resistance and MTCO1 expression in T24‐CIS cells in orthotopic BCa models (Figure S9I,J, Supporting Information).

On the other hand, we constructed shRNA‐insensitive TACO1 expression plasmid (Ins‐TACO1‐Flag) and its mutation forms, including Ins‐TACO1 (R180A)‐Flag, Ins‐TACO1 (R196A)‐Flag and Ins‐TACO1 (Q275A)‐Flag. Remarkably, the expression of Ins‐TACO1‐Flag, Ins‐TACO1 (R180A)‐Flag, and Ins‐TACO1 (Q275A)‐Flag, but not Ins‐TACO1 (R196A)‐Flag, restored the expression of MTCO1 that was reduced by TACO1 knockdown (Figure 7D; Figure S9K, Supporting Information). Consistently, the R180A and Q275A mutations in Ins‐TACO1, but not the R196A mutation, restored the complex IV activity, OCR, mtROS, stemness, cisplatin resistance, and SOX2 expression levels that were reduced by TACO1 knockdown (Figure 7E,F; Figure S9L–Q, Supporting Information). In line with our in vitro results, data from orthotopic BCa models using T24‐CIS cells showed that the R180A and Q275A mutations in Ins‐TACO1, but not the R196A mutation, restored MTCO1 expression levels and cisplatin resistance following TACO1 knockdown (Figure 7G,H; Figure S9R, Supporting Information).

Overall, these findings demonstrated the essential role of the intact TACO1‐circFOXK2‐HSP90β ternary complex in facilitating TACO1‐mediated enhancement of BCa cell stemness and cisplatin resistance through the MTCO1/OXPHOS/mtROS axis.

### 2.8 TACO1 Mitochondrial Translocation is Inversely Related to Cisplatin Treatment Response in BCa Patients

**Figure 8:**
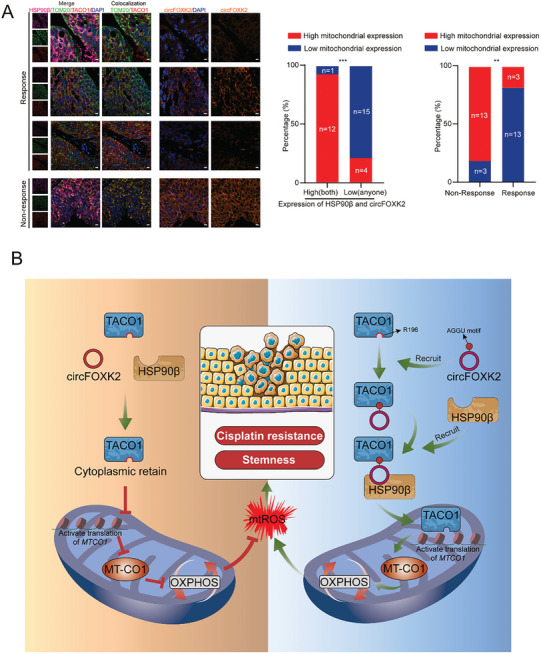
Mitochondrial TACO1 expression is inversely related to cisplatin treatment response in BCa patients. A) Representative multiplex immunohistochemistry images of tissue sections showed the expression levels of HSP90β, circFOXK2 and distribution of TACO1 in responders and non‐responders to cisplatin treatment (Left). Scale bar, 10 µm. (Middle) The correlation between the expression levels of HSP90β/circFOXK2 and mitochondrial TACO1 expression levels (Right). The correlation between cisplatin treatment response rate and mitochondrial TACO1 expression levels (Right). R: response; NR: non‐response. Image J software was used to calculate the colocalization coefficient of TACO1 and TOM20. High or low mitochondrial expression was classified based on the median colocalization coefficient across all samples (**p < 0.01, ***p < 0.001, Fisher's exact test). B) The schematic illustration showed that TACO1 translocates to mitochondria with the assistance of HSP90β and circFOXK2, subsequently enhancing OXPHOS. This enhancement, in turn, leads to increased cancer stemness, thereby contributing to cisplatin resistance in BCa cells.

Finally, we investigated whether mitochondrial TACO1 can predict the efficacy of cisplatin treatment in BCa patients. To this end, we employed multiplex immunohistochemistry (mIHC) to assess the expression levels of HSP90β, circFOXK2, and TACO1 in tissues from BCa patients who either responded or did not respond to cisplatin treatment prior radical cystectomy. We found that in patients with high expression of both HSP90β and circFOXK2, TACO1 primarily localized in the mitochondria. In contrast, in patients with low expression of HSP90β and/or circFOXK2, TACO1 primarily resided in the cytoplasm. Notably, non‐responsive BCa patients exhibited significantly higher mitochondrial TACO1 expression compared to their responsive counterparts (**Figure** **8**A). These results indicate that the expression of HSP90β and circFOXK2 facilitates mitochondrial translocation of TACO1 in BCa patients. Moreover, the presence of TACO1 in the mitochondria is associated with a diminished response to cisplatin treatment in these patients.

## 3 Discussion

Chemoresistance in cancer cells have been linked to OXPHOS hyperactivity,^[^ ^8^, ^21^ ^]^ yet the mechanism in BCa is elusive. This study shows that increased TACO1 expression in BCa enhances MTCO1 translation, boosting complex IV activity, OXPHOS, and mtROS, which in turn raises stemness and cisplatin resistance. This process relies on TACO1 translocating to the mitochondria, aided by HSP90β, with circFOXK2 acting as a scaffold for their interaction (Figure 8B). Consequently, disrupting the TACO1‐circFOXK2‐HSP90β complex inhibits cancer stemness and cisplatin resistance in BCa cells by suppressing the MTCO1/OXPHOS/mtROS axis. Clinically, BCa patients with high mitochondrial TACO1 expression respond poorly to cisplatin treatment.

CSCs represent a unique subpopulation of cancer cells characterized by various malignant phenotypes such as enhanced tumorigenicity, invasiveness, and especially, resistance to anticancer therapies. The inherent resistance of CSCs to anticancer agents, including chemotherapeutic drugs, poses a substantial challenge to effective cancer treatment. Consequently, targeting and eradicating CSCs holds significant promise in overcoming chemoresistance. The maintenance of cancer stemness is associated with oncogenic pathways like Notch, Hedgehog, Wnt, and NF‐κB.^[^ ^22^ ^]^ Notably, metabolic reprogramming, such as OXPHOS hyperactivity, is linked to cancer stemness and chemoresistance in various malignancies,^[^ ^6^, ^7^ ^]^ which we have confirmed in BCa cells.

The mitochondrial OXPHOS process, driven by five enzyme complexes within the electron transport chain, is a crucial pathway for ATP production in eukaryotic cells.^[^ ^23^ ^]^ Dysregulation of OXPHOS is associated with diseases like human mitochondrial disorders and cancer.^[^ ^24^ ^]^ While upstream regulators such as MYC, PGC1α, and PINK1 are known for their role in enhancing OXPHOS to support cancer stemness and drug resistance,^[^ ^6^, ^25^ ^]^ the precise mechanisms related to mitochondrial respiratory chain complex assembly factors remain unclear. Limited studies suggest that dysregulation of mitochondria complex assembly contributes to the progression of cancer.^[^ ^11^, ^12^ ^]^ Consistent with these researches, we unveiled a novel function of TACO1, a complex IV assembly factor, in promoting stemness and cisplatin resistance in BCa cells.

ROS elevation is recognized as a common outcome of the OXPHOS process. ROS plays dual roles in regulating cancer cell stemness and drug resistance, depending on the cancer type. In hepatocellular carcinoma, glutaminase 1 enhances stemness by reducing ROS.^[^ ^26^ ^]^ Conversely, in breast cancer, lowering ROS levels diminishes cancer cell stemness by downregulating HIF2α.^[^ ^27^ ^]^ These results suggest that ROS can either promote or inhibit cancer cell stemness by disrupting intracellular ROS homeostasis or triggering different cascade signaling responses. In BCa cells, our findings demonstrate that TACO1‐mediated OXPHOS increases mtROS, a major source of intracellular ROS, thereby promoting cancer cell stemness and cisplatin resistance via SOX2 upregulation. Previous studies have indicated that mtROS promotes protein oxidation through oxidants such as H2O2, thereby regulating signal transduction.^[^ ^28^ ^]^ However, mtROS induced by TACO1 may not directly oxidize the SOX2 protein; rather, they might target its upstream regulators, as SOX2 oxidation leads to its inactivation.^[^ ^29^ ^]^ Indeed, mtROS‐mediated oxidation of PTEN has been reported to promote its inactivation, which subsequently activates PI3K/Akt signaling, an upstream regulator of SOX2.^[^ ^30^ ^]^ Nevertheless, further investigation is needed to understand how mtROS regulates BCa stemness through SOX2.

Prior research linked TACO1 deficiency to mitochondrial dysfunction and human mitochondrial disorders,^[^ ^16^, ^19^, ^31^ ^]^ but its involvement in cancer was unexplored. Our findings align with existing evidence,^[^ ^16^ ^]^ revealing that TACO1 boosts OXPHOS by enhancing MTCO1 expression and complex IV activity in BCa cells. MTCO1 is a mitochondrially encoded gene, and its translation occurs within the mitochondria.^[^ ^32^ ^]^ However, TACO1, encoded by a nuclear gene, is translated in the cytoplasm, making it unclear how it translocates to the mitochondria. Our data from the TACO1 interactome and subsequent validation experiments confirm that HSP90β, a molecular chaperone involved in protein mitochondrial translocation,^[^ ^20^ ^]^ facilitates the mitochondrial translocation of TACO1 into the mitochondria. Intriguingly, we found that HSP90β‐mediated translocation of TACO1 requires the involvement of circFOXK2, shedding light on the RNA‐binding protein function of TACO1 and further affirming the role of circRNAs in mitochondrial translocation of cytoplasmic protein.^[^ ^33^ ^]^

Mitochondrially localized circRNAs often regulate mitochondrial function. For instance, circSamd4 promotes Vcp mitochondrial translocation to reduce mtROS in cardiomyocyte cells,^[^ ^34^ ^]^ and circPUM1 enhances OXPHOS by maintaining mitochondrial homeostasis in esophageal squamous cell carcinoma.^[^ ^34^ ^]^ Interestingly, the mitochondrial localization of circFOXK2 observed in BCa cells contrasts with its previously reported nuclear or cytosolic localization.^[^ ^35^ ^]^ In BCa cells, circFOXK2 acts as a scaffold for TACO1‐HSP90β interaction, facilitating the mitochondrial translocation of TACO1. This process, in turn, promotes cancer stemness and cisplatin resistance by enhancing the MTCO1/OXPHOS axis. This provides novel insights into our understanding of how HSP90β and mitochondrial circRNAs promote cytoplasmic protein mitochondrial translocation. Consequently, the development of nucleic acid drugs targeting circFOXK2 may present a promising approach for mitigating cisplatin resistance in BCa by disrupting the TACO1/OXPHOS axis.

In conclusion, TACO1, with circFOXK2 as a scaffold, interacts with HSP90β to translocate to mitochondria, where it boosts MTCO1 expression and OXPHOS. This process ultimately leads to increased cancer stemness, contributing to cisplatin resistance in BCa cells. Thus, mitochondrial TACO1 could serve a biomarker for cisplatin response, and targeting the TACO1‐circFOXK2‐HSP90β complex may provide a precision therapy against cisplatin resistance in BCa treatment.

## 4 Experimental Section

### Patient Information

The study was approved by the SYSUCC Institutional Review Board. Patients in the study signed the informed written consent forms based on the Declaration of Helsinki. All studies involving human participants were conducted in a blinded manner.

All tissue samples were obtained from SYSUCC and were pathologically confirmed as BCa. BCa grade and TMN stage were defined according to previous studies.^[^ ^36^ ^]^ The time from surgery to death was defined as OS. Cisplatin treatment response was evaluated according to RECIST 1.1 criteria.^[^ ^37^ ^]^

### Plasmid Construction and RNA Interference

Overexpression plasmids were constructed by cloning the corresponding sequences of full‐length TACO1, TACO1‐mutation and full‐length HSP90β into pLVX‐puro‐flag or pLVX‐blasticidin‐myc. The sequence of the MTCO1 gene was translated to the nuclear code and cloned and inserted into the pCMV‐mito plasmid. The circFOXK2 and the circFOXK2‐TACO1 binding site mutation plasmids were obtained from Genecreate (Wuhan, China).

The interference sequences for TACO1, circFOXK2, and HSP90β were cloned and inserted into pLKO.1‐puro or pLKO.1‐neo. The shRNA interference sequences are listed in Table S4, Supporting Information.

### Cell Lines

SV‐HUC‐1, 5637, J82, UMUC3, TCC‐SUP, T24, and 293T cell lines were obtained from the American Type Culture Collection. 5637 and T24 cells were cultured with RPMI 1640 medium (Invitrogen, Carlsbad, USA). UMUC3 cells were cultured in MEM basic medium (Invitrogen) supplemented with MEM NEAA (Invitrogen). Other cell lines were cultured in DMEM (Invitrogen). All media were supplemented with 10% fetal bovine serum (HyClone, USA) and 1% penicillin/streptomycin. All used cell lines were authenticated before the study began.

### Quantitative Real‐Time Polymerase Chain Reaction (qRT‐PCR)

TRIzol (Invitrogen) was used to extract total RNA from cells. PrimeScript RT Master Mix (Takara, Beijing, China) was used to synthesize cDNA. SYBR Green SuperMix (Roche, Basel, Switzerland) was used to perform qRT‐PCR. The primer sequences used in the research are listed in Table S5, Supporting Information.

### RIP and RIP‐Seq

RIP was performed according to the RIP kit protocol (Millipore, Massachusetts, USA). After centrifugation of the cell lysate, the supernatant was incubated with antibodies and magnetic beads at 4 °C overnight. After washing the beads with RIP wash buffer, TRIzol was used to extract the RNAs bound to the beads, which were then subjected to RNA sequencing or qRT‐PCR analysis. The NEB Next Ultra Directional RNA Library Prep Kit was used to construct RNA libraries, and then, the libraries were sequenced on the Illumina HiSeq platform (Illumina, California, USA). To detect circRNAs, the algorithms CIRI2 and CIRCexplorer2 were used.^[^ ^38^ ^]^ CircRNAs identified by both algorithms were classified as bound circRNAs. To map reads to the human reference genome GRCh37/hg19, BWA‐MEM, or TopHat was utilized.^[^ ^39^ ^]^

### co‐IP), Silver Staining, and MS

For co‐IP, protein extracts were obtained using lysis buffer. Protein extracts were incubated with antibodies in conjunction with protein A/G magnetic beads (Thermo Fisher, Waltham, USA) overnight at 4 °C. Proteins bound to the beads were eluted using SDS buffer and then subjected to SDS‐PAGE. The protein‐containing gel slices were subjected to MS analysis (Wininnovate Bio, Shenzhen, China) using the Easy nLC 1200 (Thermo Scientific, Waltham, USA).

### GST Pull‐Down Assays

Purification of GST‐tagged TACO1 and His‐tagged HSP90β proteins was performed using the GST‐tag Protein Purification Kit and His‐tag Protein Purification Kit (Beyotime). The GST‐TACO1 or His‐HSP90β fusion proteins were expressed and extensively amplified in BL21‐DE3 *E. coli*. The bacteria were thoroughly lysed and incubated with tag Purification Resin at 4 °C for 1 h to generate GST or His conjugates. The subsequent GST pull‐down was conducted according to the instructions provided in the GST Protein Interaction Pull‐Down kit (Thermo Fisher).

### RNA Pull‐Down

A biotin‐labeled circFOXK2 probe was created based on the junction sequence. Cell lysate, the RNA probe, and streptavidin‐coated magnetic beads (Invitrogen) were incubated overnight at 4 °C. qRT‐PCR analysis was then used to measure circFOXK2 enrichment and western blot assessed proteins bound to the beads.

CircFOXK2 junction probe (Umine‐Bio, Guangzhou, China):

5′‐biotin labeling‐GUGCUCGGGAACCUGAAUGUGCACCUUCGGGCUGUCUCCACCUGAA‐Cy5‐3′

Control probe (Sangon Biotech, Shanghai, China):

5′‐biotin labeling‐UUGUACUACACAAAAGUACUG‐3′

### IF and FISH

Cells were fixed with 4% paraformaldehyde for 15 min and penetrated with 0.5% Triton X‐100 for 10 min, followed by incubation with primary antibodies overnight at 4 °C. On the second day, the cells were incubated with ALEXA FLUOR 488 or 555 antibodies (Invitrogen) at room temperature (RT) for 1 hour in the dark. Next, the cells were incubated with 15% formamide/1×SSC for 15 min at RT. The probe was diluted with 15% formamide/1× SSC, and then 200 µL was added to the cells, which were then incubated overnight at 37 °C. For mitochondrial staining, cells were treated with MitoTracker (C1035, 1:1000 dilution, Beyotime) before fixation. Finally, an antifade mounting medium with DAPI (Beyotime) was added, and images were acquired with an LSM880 fluorescence microscope (Zeiss, Oberkochen, Germany).

### Mitochondrial Extraction

The mitochondrial extraction process was carried out following the instructions of the Cell Mitochondria Isolation Kit (Beyotime). Cells were lysed with mitochondrial isolation reagent and homogenized about 30 times. After centrifugation at 1000 × *g* for 10 min at 4 °C, the supernatant was transferred to a new tube and centrifuged again at 3500 × *g* for 10 min at 4 °C. The supernatant was discarded, leaving the precipitates as the mitochondrial fractions.

### Mitochondrial Respiratory Complex IV Activity Assay

Mitochondria were isolated using the Cell Mitochondria Isolation Kit (Beyotime). The concentration of the mitochondrial samples was determined using the Bradford Protein Assay Kit (Beyotime). The activity of mitochondrial respiratory complex IV was detected using the Mitochondrial complex IV Activity Assay Kit (Abbkine, Wuhan, China).

### CCK‐8 Assay and IC50 Assessment

For the CCK‐8 assay, around 1000 cells were seeded per well in a 96‐well plate. After cell adhesion, the medium was replaced with FBS‐free medium containing 10% CCK‐8 (APExBIO, Houston, USA) and incubated for 2 h at 37 °C with 5% CO_2_. Absorbance was measured at 450 nm.

For IC50 determination, cells were seeded in 96‐well plates and grown to 50% confluence. Medium with varying concentrations of cisplatin (0.1 to 28 µm) was added. After 48 h, the CCK‐8 assay was conducted as described. GraphPad Prism 8.0 was used to calculate the IC50.

### Colony Formation Assay

For the colony formation assay, 500 cells were seeded into each well of a six‐well plate and cultured in a complete medium for 1 to 2 weeks. The medium was then aspirated, and the cells were washed three times with PBS. They were subsequently fixed with 4% paraformaldehyde for 15 min, stained with crystal violet for another 15 min, and finally counted.

### Tumor Sphere‐Forming Assay

One BCa cell was seeded into each well of 96‐well ultralow attachment plates (Corning, New York, USA) and DMEM/F12 with 20 ng mL^−1^ EGF (Invitrogen), 10 µg mL^−1^ FGF (Invitrogen), 1× B27 (Invitrogen) and 10% BSA (Invitrogen) was added. After 7–14 days of incubation at 37 °C with 5% CO_2_, the tumor spheres were observed with an inverted microscope (Olympus, Japan).

### Mitochondrial ROS Detection and Flow Cytometry

For CD44 expression analyses, viable cells (10^6^ cells/mL) were incubated at 4 °C for 30 min with anti‐CD44/APC (BD Pharmingen, New Jersey, USA) and washed twice with PBS. Samples were then processed using a CytoFLEX Flow Cytometer (Beckman, Indianapolis, USA). Data were analyzed using CytExpert 2.2 software.

The ALDEFLUOR kit (Stem Cell Technologies, Vancouver, Canada) was used to measure ALDH1 expression as per the manufacturer's instructions. Cells were washed with PBS, centrifuged at 250 × *g* for 3 min, and the supernatant discarded. The cells were then resuspended in 500 µL of ALDEFLUOR Assay Buffer, mixed with 5 µL of activated ALDEFLUOR reagent, and incubated at 37 °C in the dark for 30 min. After centrifuging at 250 × g for 5 min and discarding the supernatant, the cells were resuspended in 500 µL of ALDEFLUOR Assay Buffer for detection.

For mtROS analyses, cells were incubated with MitoSOX (Invitrogen) at a final concentration of 5 µM at 37 °C for 10 min in the dark. Subsequently, the cells were washed three times with PBS. CytoFLEX Flow Cytometer (Beckman) was used to detect the mtROS levels in each group.

### Generation of Cisplatin‐Resistant BCa Cell Lines

To obtain cisplatin‐resistant BCa cell lines, the parental cells were initially cultured in medium supplemented with 1 µM cisplatin. As they adapted, the cisplatin concentration was gradually increased by 1 µm increments until higher tolerance was achieved, with cisplatin continuously added throughout the process. The IC50 value was used to assess the resistance of BCa cells to cisplatin.

### ECAR and OCR Measurement

ECAR and OCR were measured using the Agilent Seahorse XF Glycolysis Stress Test Kit (Agilent, California, USA) and the Agilent Seahorse XF Cell Mito Stress Test Kit (Agilent), respectively, following the respective instructions. First, each kind of cell was evenly inoculated in the XF96 cell culture microplates.

For the ECAR assay, cells were incubated in Seahorse XF base medium with 2 mm glutamine at 37 °C in a non‐CO^2^ incubator for 1 h. During this time, 10 mm glucose, 1 µm oligomycin, and 50 mm 2‐DG were added. The ECAR values were then measured using the analyzer.

For the OCR assay, cells were incubated in Seahorse XF base medium (containing 1 mm pyruvate, 2 mm glutamine, and 10 mm glucose) at 37 °C in a non‐CO^2^ incubator for 1 h. During this time, 0.5 µm each of oligomycin, FCCP, antimycin A, and rotenone were added. OCR values were then measured using the analyzer.

### Polysome Profiling

Polysome profiling was performed as previously described.^[^ ^40^ ^]^ Briefly, the cells were lysed and centrifuged, and the supernatant was collected. Gradient buffer was used to configure a sucrose solution with a concentration gradient of 10–50%, and the cell lysate was placed in the uppermost layer of the sucrose gradient and then centrifuged at 36 000 rpm for 2 h at 4 °C with a Beckman SW41Ti rotor (Beckman). A UA‐6 absorbance detector was used to record the polysome profiles and the absorbance was measured at 260 nm. RNA from each fraction was subsequently extracted with TRIzol.

### Glycerol Gradient Sedimentation

Glycerol gradient sedimentation was performed as previously reported.^[^ ^41^ ^]^ Briefly, after lysing the cells with the lysis buffer (HEPES 10 mm, MgCl_2_ 2 mm, KCl 10 mm, NP‐40 0.05%, EDTA 0.5 mm, NaCl 150 mm, DTT 1 mm, PMSF 0.1% and 1 × protease cocktail), the lysates were layered on a 5–25% gradient glycerol solution. Gradients were centrifuged for 16 h at 4 °C and 36000 rpm using an SW41 rotor (Beckman) and fractionated from the top of the centrifugation tubes. Extraction of protein from glycerol solution was performed using methanol and chloroform methods. Related proteins were detected by western blot.

### Animal Model

The animal ethics committee of SYSUCC approved this study. The operations adhered to both SYSUCC‐approved guidelines and ethical regulations for animal studies. Female BALB/c nude mice (4–5 weeks old) used in this study were purchased from Guangdong Medical Laboratory Animal Center.

For the tumor‐initiating capacity assay, the appropriate number of cells was injected subcutaneously into BALB/c nude mice, and tumor formation was observed after 2 months. The proportion of tumor‐initiating cells was estimated using extreme limiting dilution analysis.^[^ ^42^ ^]^

For the bladder orthotopic xenograft model, isoflurane was used for the maintenance of anesthesia in mice. A 24 G closed IV catheter was inserted into the urethras of the mice. Residual urine was withdrawn from the bladder and then the bladder was irrigated with 0.1 m HCl, 0.1 m NaOH, and PBS in turn. Next, 1 × 10^6^ cells stably expressing the luciferase were injected into the bladder and retained for 30 min. When the tumors in both groups reached the same size, cisplatin was administered intraperitoneally at 2 mg kg^−1^ to one mouse every 3 days for a total of 10 doses. Finally, after the experimental procedures, the mice were sacrificed by cervical dislocation, and their bladders were excised. Hematoxylin‐eosin staining was used to assess the size of tumors in the bladder. IHC was used to evaluate the expression levels of related proteins in tumor tissues. Tumor volume in the bladder was dynamically observed using the IVIS 200 imaging system, with intraperitoneal injection of potassium D‐fluorescein (Goldbio, USA) conducted before the observation.

### Molecular Docking

The HDOCK server was used to perform molecular docking among circFOXK2, TACO1, and HSP90β.^[^ ^43^ ^]^ The 3D structure of TACO1 (predicted by AlphaFold2), the crystal structure of HSP90β (PDB code: 7Z38), and the 3D structure of circFOXK2 (predicted by RNA composer) were used in the docking simulation.^[^ ^44^ ^]^ The HDOCK server automatically predicts their interaction through a hybrid algorithm of template‐based and template‐free docking. Molecular graphics were generated by PyMOL.

### IHC

IHC was performed using the standard streptavidin‐biotin peroxidase complex method as previously described.^[^ ^45^ ^]^ Briefly, tissues were dewaxed with xylene, and rehydrated with ethanol, and endogenous peroxidase was blocked using 3% hydrogen peroxide. Antigen retrieval was done with EDTA (pH 8.0). After blocking with 5% BSA for 1 h at RT, primary antibodies were incubated overnight at 4 °C. The next day, tissues were washed with PBS and incubated with goat anti‐mouse/rabbit IgG polymer for 1 h at RT. DAB and hematoxylin staining were then performed. The staining index is divided into four levels: negative (0), weakly (1), moderate (2), and strong (4), and the positive staining area score was also divided into four levels, <10% (1), 10–40% (2); 40–70% (3); and >70% (4). The product of the staining index and the positive area score was defined as the immunoreactivity score (IS) (0‐4, low expression; 6–12, high expression). The IS of each tissue section was assessed by two independent clinical pathologists.

### mIHC

According to the manufacturer's instructions (Absin, Shanghai, China), slides were dewaxed with xylene, rehydrated with ethanol, and incubated with 3% hydrogen peroxide for 10 min to block endogenous peroxidase. Antigen retrieval was done using EDTA (pH 8.0). After cooling to RT, tissue sections were incubated with 5% BSA for 1 h to block non‐specific binding. The primary antibody solution was added, and sections were incubated overnight at 4 °C. The next day, tissues were washed with PBS‐T three times. Sections were then incubated with HRP‐conjugated anti‐rabbit or anti‐mouse IgG at RT for 10 min, followed by a 10 min reaction with TSA reagents conjugated to fluorophores (PPD 520, PPD 570, or PPD 620). After another PBST wash, antigen retrieval using EDTA (pH 8.0) was repeated, followed by the next staining round.

### Antibodies

For western blot: anti‐TACO1 (A15445, 1:1000 dilutions, Abclonal, Wuhan, China), anti‐MTCO1 (62 101, 1:1000 dilution, Cell Signaling Technology, Boston, USA), anti‐COX IV (4850 1:1000 dilutions, Cell Signaling Technology), anti‐ATP5A (ab176569, 1:1000 dilution, Abcam, Cambridge, UK), anti‐NDUFB8 (ab192878, 1:1000 dilution, Abcam), anti‐COX II (ab79393, 1:1000 dilution, Abcam), anti‐SDHB (ab178423, 1:1000 dilution, Abcam), anti‐UQCRC2 (ab203832, 1:1000 dilution, Abcam), anti‐HSP90β (11405‐1‐AP, 1:1000 dilution, Proteintech, Chicago, USA), anti‐Histone H3 (60 932, 1:5000 dilution, Cell Signaling Technology), anti‐α‐tubulin (66031‐1‐Ig, 1:10 000, dilutions, Proteintech), anti‐Flag (8146, 1:1000 dilution, Cell Signaling), anti‐KLF4 (11880‐1‐AP, 1:5000 dilution, Proteintech), anti‐OCT4 (11263‐1‐AP, 1:5000 dilution, Proteintech), anti‐c‐Myc (10828‐1‐AP, 1:5000 dilution, Proteintech), anti‐SOX2 (23064S, 1:2000 dilution, Cell Signaling Technology).

For IP, RIP and IHC: anti‐HSP90β (11405‐1‐AP, 1:200 dilution, Proteintech), anti‐TACO1 (21147‐1‐AP, 1:200 dilution, Proteintech), anti‐Flag (8146, 1:100 dilution, Cell Signaling). Anti‐MTCO1 (A24805, 1:200 dilution, Abclonal, Wuhan, China).

For IF: anti‐TACO1 (sc‐398915, 1:50 dilution, Santa Cruz, Dallas, USA), anti‐TOM20 (11802‐1‐AP, 1:200 dilution, Proteintech), anti‐Flag (8146, 1:200 dilution, Cell Signaling Technology),

### Statistical Analysis

An independent samples t‐test, χ^2^ test, and Fisher's exact test were used to identify differences between the two groups. Survival differences were assessed with Kaplan‐Meier curves and Cox hazard analysis. X‐tile was used to determine the optimal cut‐off value. Statistical significance was set at *p* < 0.05. Analyses were conducted using SPSS 26.0 or GraphPad Prism 8.0. Data are presented as mean ± standard deviation (SD), with experiments independently repeated at least three times.

## Conflict of Interest

The authors declare no conflict of interest.

## Author Contributions

M.‐H.D., Z.‐H.Z., J.‐W.C., and X.‐D.L. contributed equally to this work. M.‐H.D., X.‐C.Z., and Z.‐W.L. conceived this project and designed the experiments. M.‐H.D., Z.‐H.Z., and J.‐W.C. carried out the experiments with help from Z.‐F.L., N.W., W.‐S.W., Y.‐L.P., L.‐J.J., X.L., and F.‐J.Z. M.‐H.D., Z.‐H.Z., J.‐W.C., X.‐D.L., and J.‐W.Y. performed data curation and formal analysis. M.‐H.D. drafted the manuscript. X.‐C.Z. and Z.‐W.L. supervised the study and revised the manuscript. All authors have reviewed and approved the final version of the manuscript.

## Supporting information



Supporting Information

## Data Availability

The data that support the findings of this study are available in the supplementary material of this article.
