# Supplementary material for: Enhanced Oxidative Phosphorylation Driven by TACO1 Mitochondrial Translocation Promotes Stemness and Cisplatin Resistance in Bladder Cancer
Source: Adv Sci (Weinh). 2024 Dec 10;12(5):2408599. doi: 10.1002/advs.202408599 (PMC11791945; doi:10.1002/advs.202408599)
Supplement: Supplementary file 1 — Supporting Information [file ADVS-12-2408599-s001.pdf]

ADVANCED SCIENCE
Open Access

Supporting Information

for *Adv. Sci.*, DOI 10.1002/advs.202408599

Enhanced Oxidative Phosphorylation Driven by TACO1 Mitochondrial Translocation Promotes Stemness and Cisplatin Resistance in Bladder Cancer

Minhua Deng, Zhaohui Zhou, Jiawei Chen, Xiangdong Li, Zefu Liu, Jingwei Ye, Wensu Wei, Ning Wang, Yulu Peng, Xin Luo, Lijuan Jiang, Fangjian Zhou, Xianchong Zheng and Zhuowei Liu**

Supporting Information for

Enhanced Oxidative Phosphorylation Driven by TACO1 Mitochondrial Translocation Promotes Stemness and Cisplatin Resistance in Bladder Cancer

Minhua Deng[a,b,1], *Zhaohui Zhou*[a,b,1], *Jiawei Chen,*[a,b,c,1], *Xiangdong Li*[a,b,1], *Zefu Liu*[a,b], *Jingwei Ye*[a,b], *Wensu Wei*[a,b], *Ning Wang*[a,b], *Yulu Peng*[a,b], *Xin Luo*[a,b], *Lijuan Jiang*[a,b], *Fangjian Zhou*[a,b], *Xianchong Zheng*[a,b,*], *Zhuowei Liu*[a,b,d,*]

[a] State Key Laboratory of Oncology in South China, Guangdong Provincial Clinical Research Center for Cancer, Sun Yat-sen University Cancer Center, Guangzhou 510060, China.

[b] Department of Urology, Sun Yat-Sen University Cancer Center, Guangzhou 510060, China

[c] Department of Urology, Shunde Hospital, Southern Medical University (The First People's Hospital of Shunde Foshan), Foshan 528000, China.

[d] Department of Urology, Sun Yat-sen University Cancer Center Gansu Hospital, Lanzhou 730050, China.

[1] Minhua Deng, Zhaohui Zhou, Jiawei Chen and Xiangdong Li contributed equally to this work.

[*] Corresponding authors: liuzhw@sysucc.org.cn (Zhuowei Liu) and zhengxc@sysucc.org.cn (Xianchong Zheng)

Supplementary Figures

Figure S1

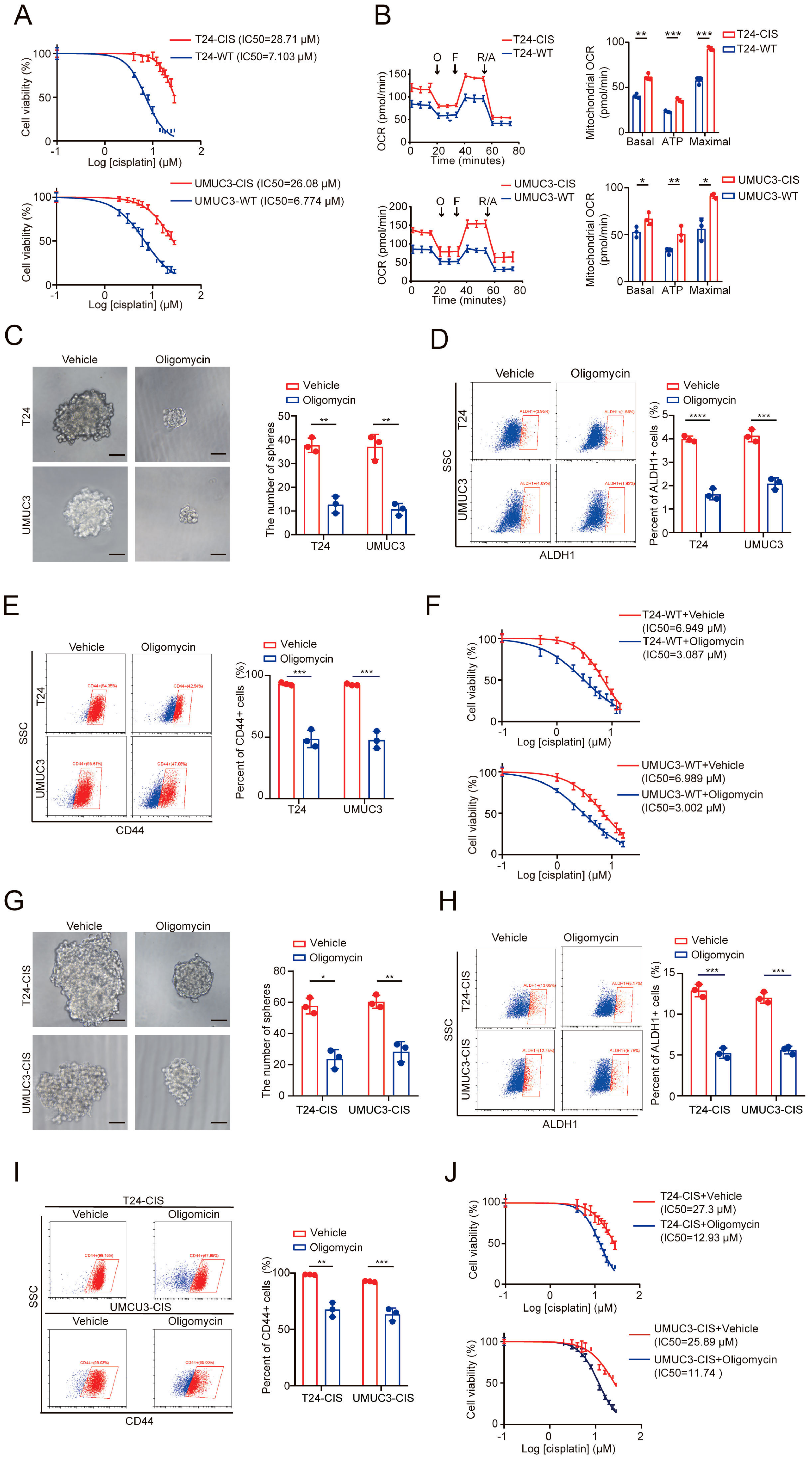
A
T24-CIS (IC50=28.71 μM)
T24-WT (IC50=7.103 μM)
UMUC3-CIS (IC50=26.08 μM)
UMUC3-WT (IC50=6.774 μM)
Cell viability (%)
Log [cisplatin] (μM)
B
T24-CIS
T24-WT
UMUC3-CIS
UMUC3-WT
O
F
R/A
OCR (pmol/min)
Time (minutes)
Mitochondrial OCR (pmol/min)
Basal
ATP
Maximal
C
Vehicle
Oligomycin
T24
UMUC3
The number of spheres
D
SSC
ALDH1
Percent of ALDH1+ cells (%)
E
CD44
Percent of CD44+ cells (%)
F
T24-WT+Vehicle (IC50=6.949 μM)
T24-WT+Oligomycin (IC50=3.087 μM)
UMUC3-WT+Vehicle (IC50=6.989 μM)
UMUC3-WT+Oligomycin (IC50=3.002 μM)
G
T24-CIS
UMUC3-CIS
H
I
UMCU3-CIS
Oligomicin
J
T24-CIS+Vehicle (IC50=27.3 μM)
T24-CIS+Oligomycin (IC50=12.93 μM)
UMUC3-CIS+Vehicle (IC50=25.89 μM)
UMUC3-CIS+Oligomycin (IC50=11.74)

Figure S1. OXPHOS promotes stemness and cisplatin resistance of BCa cells. (A) The WT or CIS BCa cells were treated with increasing concentrations of cisplatin for 48 hours. Cell viability was assessed using a CCK-8 assay, and IC50 values were calculated through nonlinear regression analysis. Data are shown as the mean ± SD, n = 3. **(B)** Detection of OCR levels in WT or CIS BCa cells by seahorse. O: Oligomycin; F: FCCP; R/A: Rotenone & Antimycin A. Data are shown as the mean ± SD, n = 3 (**p* < 0.05, ***p* < 0.01, ****p* < 0.001, independent Student's *t*-test). **(C)** The sphere-forming capacity of wild-type BCa cells treatment with vehicle or oligomycin. Scale bar, 100 μm. Data are shown as the mean ± SD, n = 3 (***p* < 0.01, independent Student's *t*-test). **(D, E)** Flow cytometry assay showed the proportion of ALDH1+ or CD44+ cells in wild-type BCa cells treated with or without oligomycin. Data are shown as the mean ± SD, n = 3 (****p* < 0.001, independent Student's *t*-test). **(F)** Corresponding wild-type BCa cells were treated with increasing concentrations of cisplatin for 48 hours. Cell viability was assessed using a CCK-8 assay, and IC50 values were calculated through nonlinear regression analysis. Data are shown as the mean ± SD, n = 3**. (G)** The sphere-forming capacity of BCa CIS cells was assessed with or without oligomycin treatment. Scale bar, 100 μm. Data are shown as the mean ± SD, n = 3 (**p* < 0.05, ***p* < 0. 01, independent Student's *t*-test). **(H, I)** Flow cytometry assay showed the proportion of ALDH1+ or CD44+ cells in cisplatin resistance BCa cells treated with or without oligomycin. Data are shown as the mean ± SD, n = 3 (****p* < 0.001, independent Student's *t*-test). **(J)** The BCa CIS cells were treated with or without oligomycin and then cultured with increasing concentrations of cisplatin for 48 hours. Cell viability was assessed using a CCK-8 assay, and IC50 values were calculated through nonlinear regression analysis. Data are shown as the mean ± SD, n = 3**.**

Figure S2

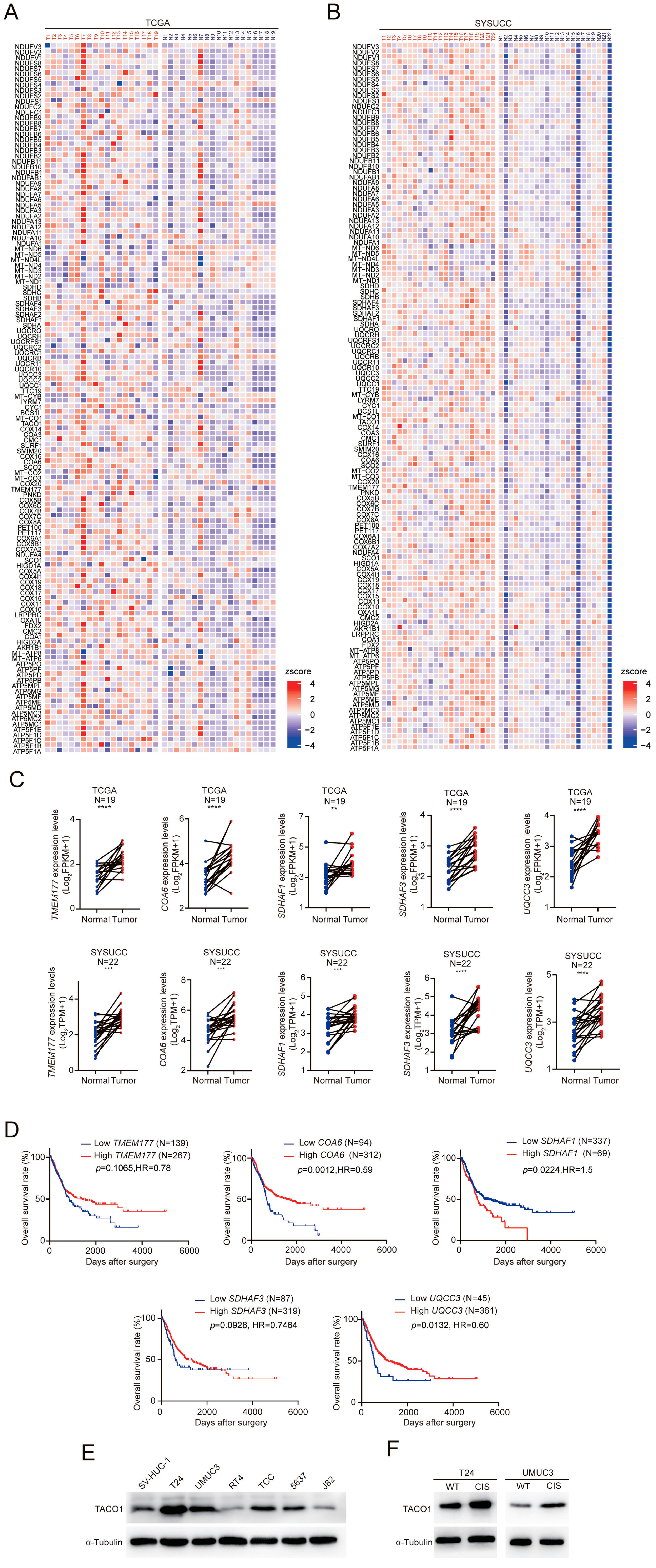
A
TCGA
B
SYSUCC
zscore
C
TCGA
N=19
Normal Tumor
SYSUCC
N=22
D
Overall survival rate (%)
Days after surgery
Low TMEM177 (N=139)
High TMEM177 (N=267)
p=0.1065,HR=0.78
Low COA6 (N=94)
High COA6 (N=312)
p=0.0012,HR=0.59
Low SDHAF1 (N=337)
High SDHAF1 (N=69)
p=0.0224,HR=1.5
Low SDHAF3 (N=87)
High SDHAF3 (N=319)
p=0.0928, HR=0.7464
Low UQCC3 (N=45)
High UQCC3 (N=361)
p=0.0132, HR=0.60
E
SV-HUC-1
T24
UMUC3
RT4
TCC
5637
J82
TACO1
α-Tubulin
F
T24
UMUC3
WT
CIS

Figure S2. Assembly factors of complex IV are frequently up-regulated in BCa tissues and TACO1 upregulation in other kinds of cancers from TCGA tissue. (A, B) The heatmap displays the expression profiles of 130 mitochondrial complex I-V assembly factors in the TCGA and SYSUCC of BCa patients. **(C)** The mRNA expression of *TMEM117*, *COA6*, *SDHAF1*, *SDHAF3* and *UQCC3* in SYSUCC and TCGA BCa tissue (***p <0.001, ****p <0.0001, paired sample t-test). **(D)** In the TCGA database, the expression levels of *TMEM117*, *COA6*, *SDHAF1*, *SDHAF3,* and *UQCC3* were analyzed in relation to OS in patients BCa. **(E)** The endogenous expression levels of the TACO1 protein in BCa cell lines and bladder urothelial cells (SV-HUC-1). **(F)** The endogenous expression levels of the TACO1 protein in wild-type and cisplatin-resistance BCa cell lines**.**

Figure S3

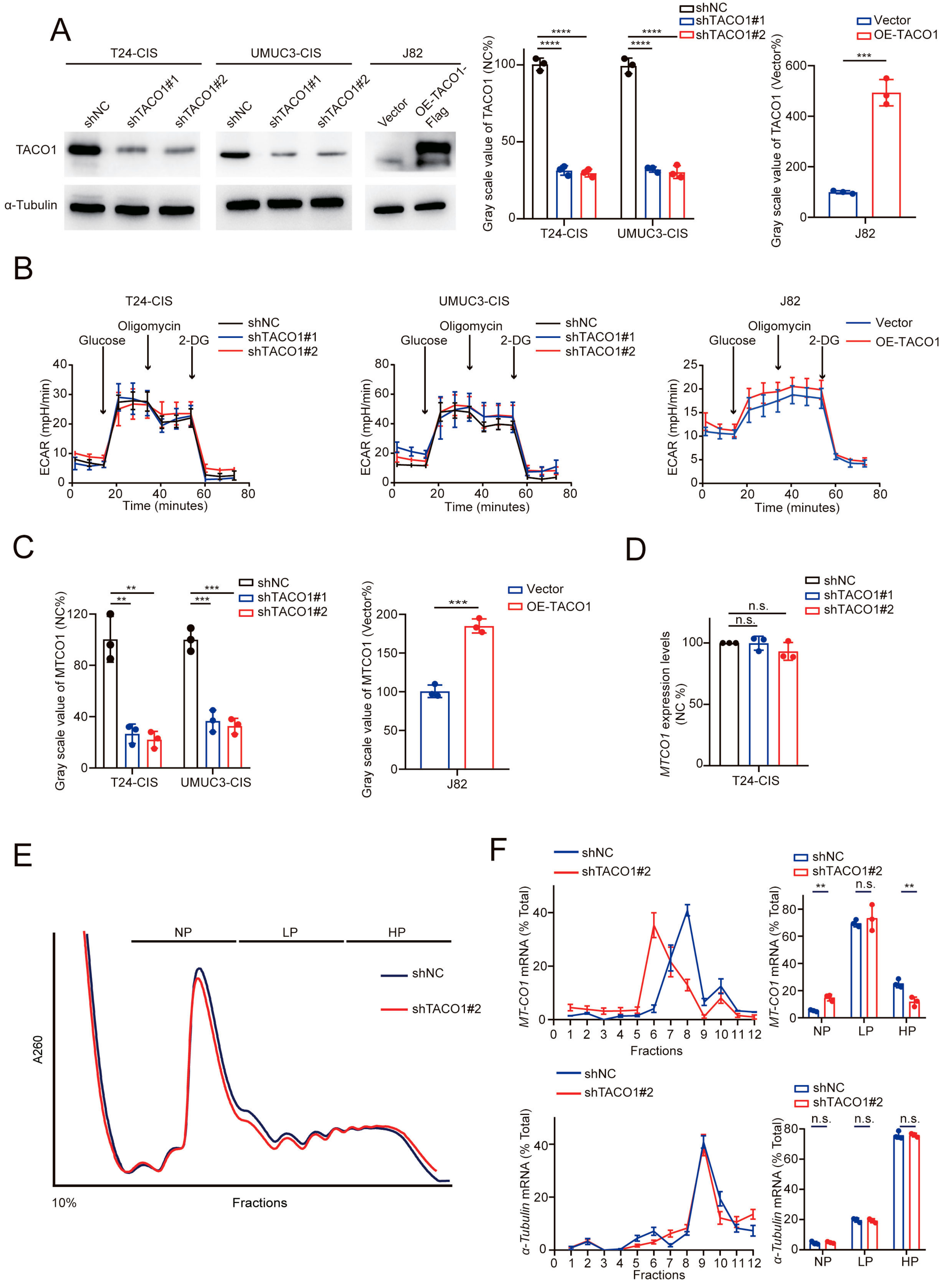
A
T24-CIS
UMUC3-CIS
J82
shNC
shTACO1#1
shTACO1#2
Vector
OE-TACO1-Flag
TACO1
α-Tubulin
Gray scale value of TACO1 (NC%)
Gray scale value of TACO1 (Vector%)
****
***
B
Glucose
Oligomycin
2-DG
ECAR (mpH/min)
Time (minutes)
OE-TACO1
C
Gray scale value of MTCO1 (NC%)
Gray scale value of MTCO1 (Vector%)
**
D
n.s.
MTCO1 expression levels (NC %)
E
NP
LP
HP
A260
10%
Fractions
F
MT-CO1 mRNA (% Total)
α-Tubulin mRNA (% Total)

Figure S3. Effects of TACO1 on translation activation and *MTCO1* expression in BCa cells (A) (Left) Western blot detected the efficiency of TACO1 knockdown or overexpression in the corresponding cell lines; (Right) Detection of the grayscale values of related proteins. Data are shown as the mean ± SD, n = 3 (****$p < 0.0001$, independent Student's *t*-test). **(B)** The seahorse analysis results showed the effect of TACO1 on ECAR in BCa cells. **(C)** Detection of the grayscale values of related proteins. Data are shown as the mean ± SD, n = 3 (**$p < 0.01$, ***$p < 0.001$, independent Student's *t*-test). **(D)** The impact of TACO1 knockdown on MTCO1 mRNA. Data are shown as the mean ± SD, n = 3 (n.s. = non-significant, independent Student's t-test). **(E)** Impact of TACO1 on polysome profiling assays. **(F)** The polysome assay was performed using T24-CIS cell lysates, the product was aliquoted into 12 fractions, and total RNA was extracted from each fraction. *MTCO1* and *α-Tubulin* mRNA expression was measured in each fraction using qRT-PCR. Fractions 1-4 were defined as non-polysomes (NP), fractions 5-8 were defined as light polysomes (LP) and fractions 9-12 were defined as heavy polysomes (HP). Data are shown as the mean ± SD, n = 3 (**$p < 0.01$, n.s. = non-significant, independent Student's *t*-test).

Figure S4

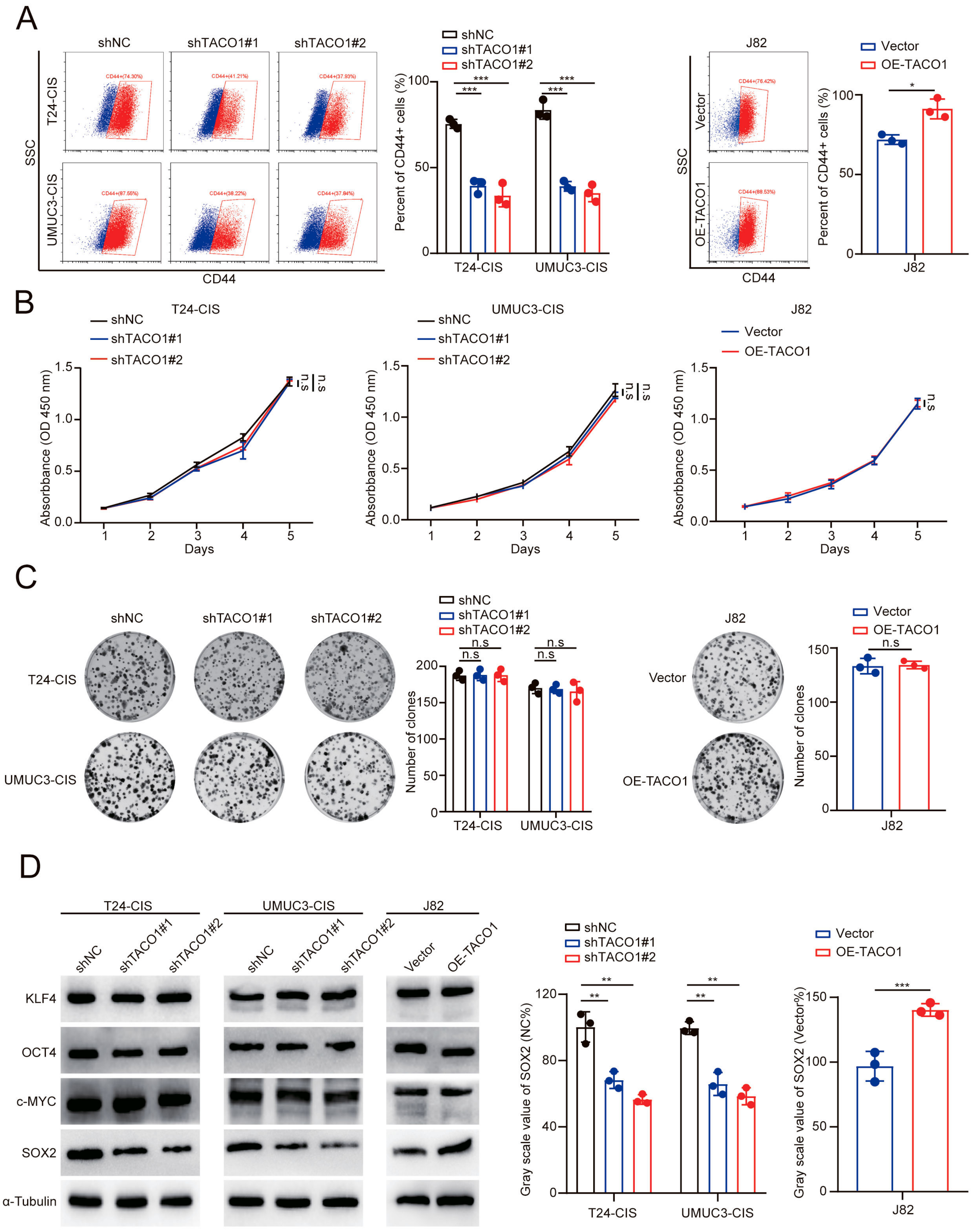

A
shNC
shTACO1#1
shTACO1#2
T24-CIS
UMUC3-CIS
SSC
CD44
Percent of CD44+ cells (%)
J82
Vector
OE-TACO1
B
T24-CIS
UMUC3-CIS
Absorbance (OD 450 nm)
Days
n.s
C
Number of clones
D
KLF4
OCT4
c-MYC
SOX2
α-Tubulin
Gray scale value of SOX2 (NC%)
Gray scale value of SOX2 (Vector%)

Figure S4. The effect of TACO1 on the proliferation ability and stemness of bladder cancer cells.

The effect of TACO1 on the proliferation ability and stemness of bladder cancer cells. (A) Flow cytometry assay showed the proportion of CD44+ cells among BCa cells. Data are shown as the mean ± SD, n = 3 (****p* < 0.001, independent Student's *t*-test). **(B)** CCK-8 assays to detect the effect of TACO1 on the proliferation ability of BCa cells. Data are shown as the mean ± SD, n = 3 (n,s. = non-significant, the two-way ANOVA). **(C)** Colony formation assays to investigate the effect of TACO1 on the proliferation ability of BCa cells. Data are shown as the mean ± SD, n = 3 (n,s. = non-significant, independent Student's *t*-test). **(D)** (Left) Western blot showed the expression of KLF4, OCT4, SOX2 and c-Myc after knockdown or overexpression of TACO1 in BCa cells. (Right) Detection of the grayscale values of related proteins. Data are shown as the mean ± SD, n = 3 (***p* < 0.01, ****p* < 0.001, independent Student's *t*-test).

Figure S5

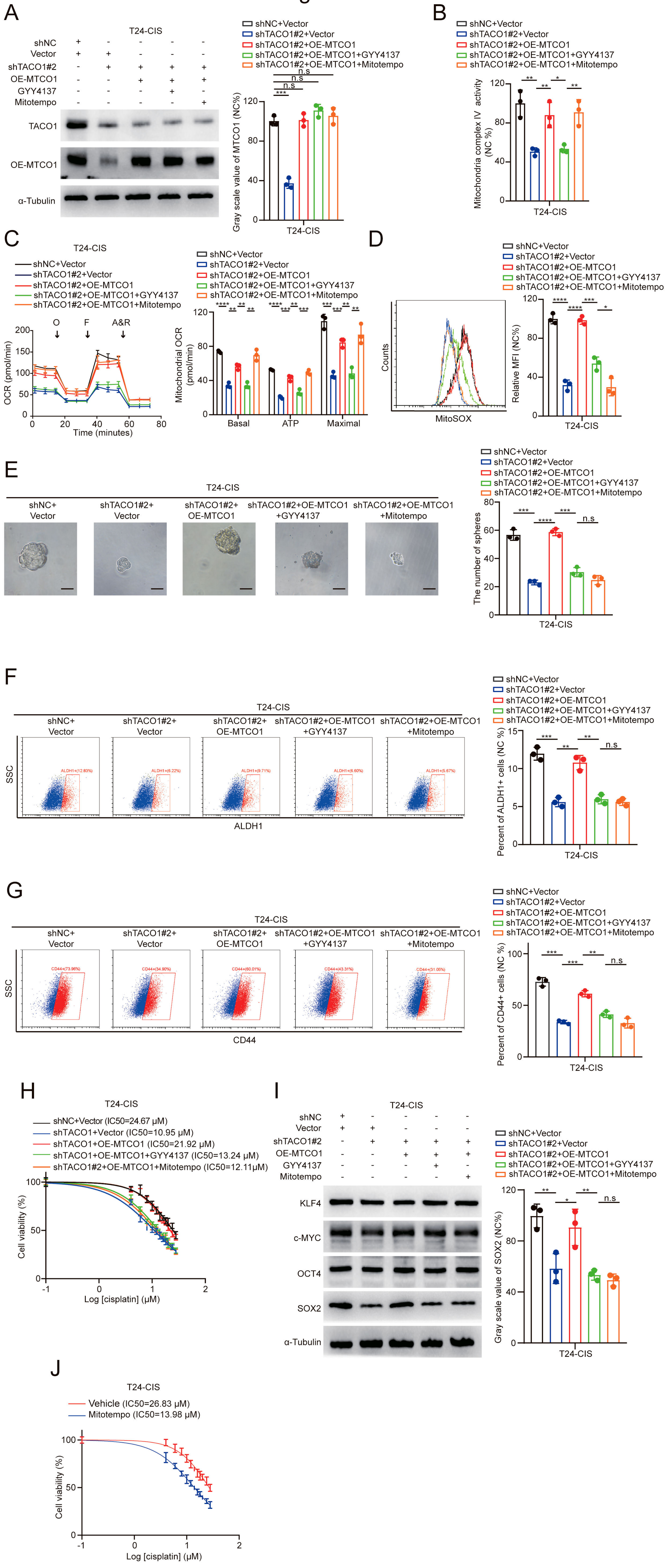
A
T24-CIS
shNC
Vector
shTACO1#2
OE-MTCO1
GYY4137
Mitotempo
TACO1
OE-MTCO1
α-Tubulin
shNC+Vector
shTACO1#2+Vector
shTACO1#2+OE-MTCO1
shTACO1#2+OE-MTCO1+GYY4137
shTACO1#2+OE-MTCO1+Mitotempo
Gray scale value of MTCO1 (NC%)
B
Mitochondria complex IV activity (NC %)
C
OCR (pmol/min)
Time (minutes)
Mitochondrial OCR (pmol/min)
Basal
ATP
Maximal
D
Counts
MitoSOX
Relative MFI (NC%)
E
shNC+ Vector
shTACO1#2+ Vector
shTACO1#2+ OE-MTCO1
shTACO1#2+OE-MTCO1 +GYY4137
shTACO1#2+OE-MTCO1 +Mitotempo
The number of spheres
F
SSC
ALDH1
Percent of ALDH1+ cells (NC %)
G
CD44
Percent of CD44+ cells (NC %)
H
shNC+Vector (IC50=24.67 μM)
shTACO1+Vector (IC50=10.95 μM)
shTACO1+OE-MTCO1 (IC50=21.92 μM)
shTACO1+OE-MTCO1+GYY4137 (IC50=13.24 μM)
shTACO1#2+OE-MTCO1+Mitotempo (IC50=12.11μM)
Cell viability (%)
Log [cisplatin] (μM)
I
KLF4
c-MYC
OCT4
SOX2
Gray scale value of SOX2 (NC%)
J
Vehicle (IC50=26.83 μM)
Mitotempo (IC50=13.98 μM)

Figure S5. TACO1 stimulates stemness and cisplatin resistance by promoting the MTCO1/OXPHOS axis in BCa cells. (A) (Left) Western blot was used to detect alterations in the TACO1 and MTCO1 protein levels in the corresponding subgroup. (Right) Detection of the grayscale values of related proteins. Data are shown as the mean ± SD, n = 3 (***$p < 0.001$, n.s. = non-significant, independent Student's *t*-test). **(B, C)** The complex IV activity and OCR levels in corresponding groups of T24-CIS cells. O: Oligomycin; F: FCCP; R/A: Rotenone & Antimycin A. Data are shown as the mean ± SD, n = 3 (*$p < 0.05$, **$p < 0.01$, ***$p < 0.001$, independent Student's *t*-test). **(D)** Flow cytometry was used to assess the levels of MitoSOX in each treatment group. Data are shown as the mean ± SD, n = 3 (*$p < 0.05$, ***$p < 0.001$, ****$p < 0.0001$, independent Student's *t*-test). **(E)** The variations in sphere-forming capacity across different treatment groups in BCa cells. Scale bar, 100 μm. Data are shown as the mean ± SD, n = 3 (*$p < 0.05$, ***$p < 0.001$, ****$p < 0.0001$, independent Student's *t*-test). **(F, G)** Flow cytometry was used to assess the percent of ALDH1+ and CD44+ in BCa cells. Data are shown as the mean ± SD, n = 3 (**$p < 0.01$, ***$p < 0.001$, n.s. = non-significant, independent Student's *t*-test). **(H)** IC50 assay to investigate the changes in cisplatin resistance among different treatment groups in BCa cells. Cellular viability was determined by CCK-8 assay, and nonlinear regression analysis was used to calculate the IC50. Data are shown as the mean ± SD, n = 3. **(I)** (Left) Western blot showed alterations in related stemness protein following different treatments in BCa cells. (Right) Detection of the grayscale values of related proteins. Data are shown as the mean ± SD, n = 3 (*$p < 0.05$, **$p < 0.01$, n.s. = non-significant, independent Student's *t*-test). **(J)** The effect of mitoTempo on cisplatin resistance in BCa cells. Cellular viability was determined by CCK-8 assay, and nonlinear regression analysis was used to calculate the IC50. Data are shown as the mean ± SD, n = 3.

Figure S6

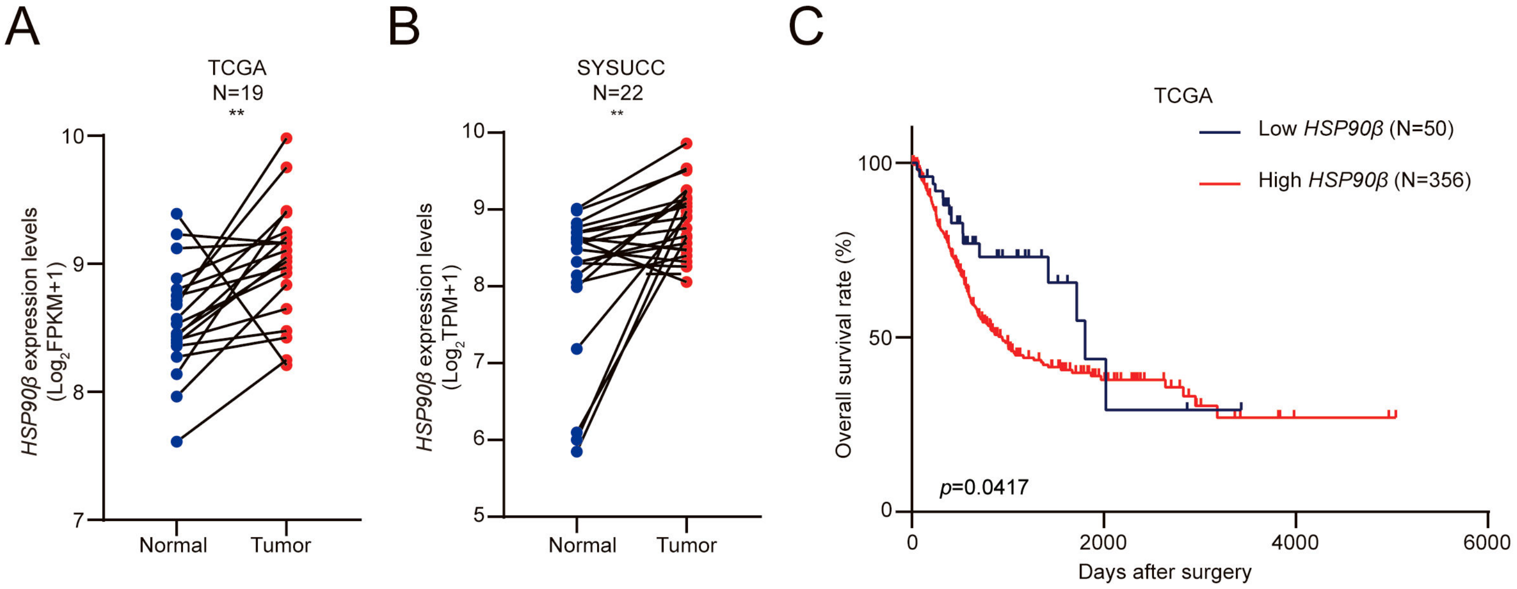
A
TCGA
N=19
**
HSP90β expression levels (Log2FPKM+1)
10
9
8
7
Normal
Tumor
B
SYSUCC
N=22
**
HSP90β expression levels (Log2TPM+1)
10
9
8
7
6
5
Normal
Tumor
C
TCGA
Low HSP90β (N=50)
High HSP90β (N=356)
Overall survival rate (%)
100
50
0
p=0.0417
0
2000
4000
6000
Days after surgery

Figure S6. HSP90β is upregulated in BCa tissue and affects the prognosis of BCa patients. (A, B) The expression levels of *HSP90β* in BCa tissue compared with matched normal tissue from TCGA and SYSUCC (**p < 0.01, paired sample t-test). **(C)** Relationship between *HSP90β* expression and BCa patient's prognosis in TCGA.

Figure S7

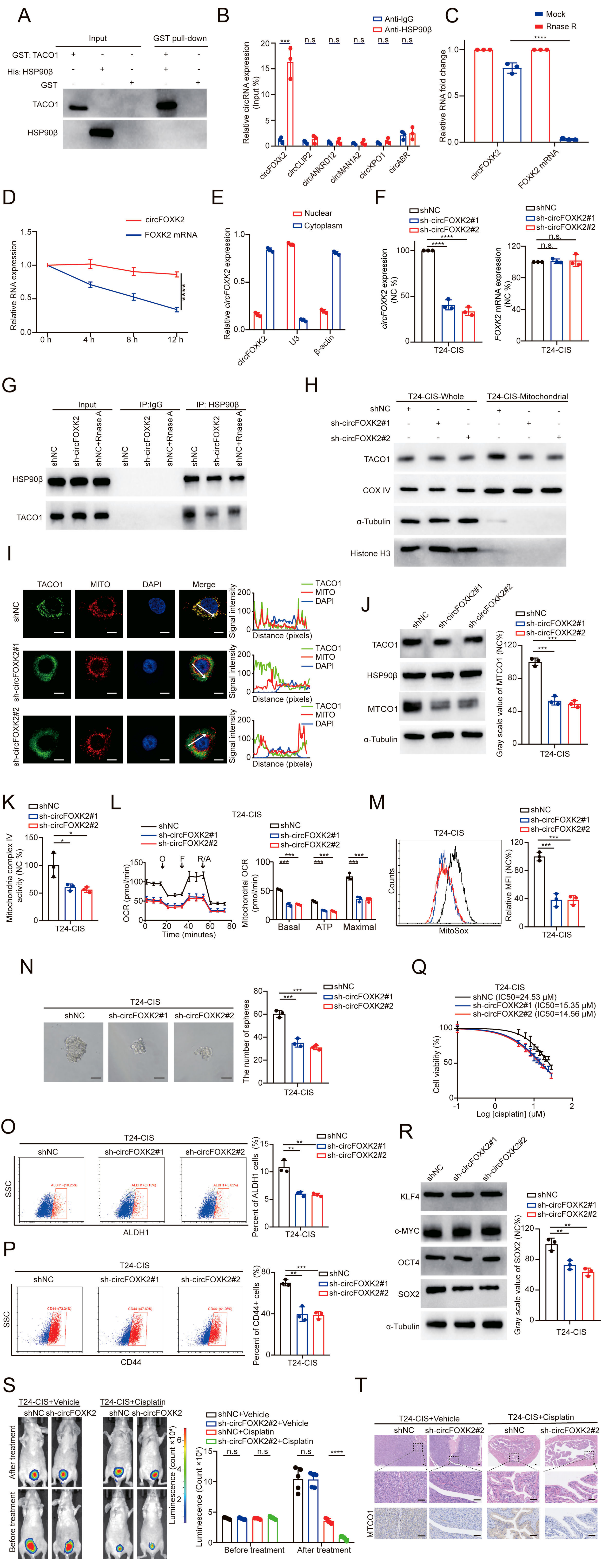

A
Input
GST pull-down
GST: TACO1
His: HSP90β
GST
TACO1
HSP90β
B
Anti-IgG
Anti-HSP90β
Relative circRNA expression (Input %)
circFOXK2
circCLIP2
circANKRD12
circMAN1A2
circXPO1
circABR
n.s
C
Mock
Rnase R
Relative RNA fold change
circFOXK2
FOXK2 mRNA
D
circFOXK2
FOXK2 mRNA
Relative RNA expression
0 h
4 h
8 h
12 h
E
Nuclear
Cytoplasm
Relative circFOXK2 expression
circFOXK2
U3
β-actin
F
shNC
sh-circFOXK2#1
sh-circFOXK2#2
circFOXK2 expression (NC %)
FOXK2 mRNA expression (NC %)
T24-CIS
n.s.
G
Input
IP:IgG
IP: HSP90β
shNC
sh-circFOXK2
shNC+Rnase A
HSP90β
TACO1
H
T24-CIS-Whole
T24-CIS-Mitochondrial
shNC
sh-circFOXK2#1
sh-circFOXK2#2
TACO1
COX IV
α-Tubulin
Histone H3
I
TACO1
MITO
DAPI
Merge
shNC
sh-circFOXK2#1
sh-circFOXK2#2
Signal intensity
Distance (pixels)
J
shNC
sh-circFOXK2#1
sh-circFOXK2#2
TACO1
HSP90β
MTCO1
α-Tubulin
Gray scale value of MTCO1 (NC%)
T24-CIS
K
Mitochondria complex IV activity (NC %)
T24-CIS
L
T24-CIS
OCR (pmol/min)
Time (minutes)
O
F
R/A
Mitochondrial OCR (pmol/min)
Basal
ATP
Maximal
M
T24-CIS
Counts
MitoSox
Relative MFI (NC%)
T24-CIS
N
T24-CIS
shNC
sh-circFOXK2#1
sh-circFOXK2#2
The number of spheres
T24-CIS
Q
T24-CIS
shNC (IC50=24.53 μM)
sh-circFOXK2#1 (IC50=15.35 μM)
sh-circFOXK2#2 (IC50=14.56 μM)
Cell viability (%)
Log [cisplatin] (μM)
O
T24-CIS
SSC
ALDH1
Percent of ALDH1 cells (%)
T24-CIS
R
KLF4
c-MYC
OCT4
SOX2
α-Tubulin
Gray scale value of SOX2 (NC%)
T24-CIS
P
T24-CIS
SSC
CD44
Percent of CD44+ cells (%)
T24-CIS
S
T24-CIS+Vehicle
T24-CIS+Cisplatin
shNC sh-circFOXK2
After treatment
Before treatment
Luminescence (count ×10⁴)
shNC+Vehicle
sh-circFOXK2#2+Vehicle
shNC+Cisplatin
sh-circFOXK2#2+Cisplatin
Luminescence (Count ×10⁶)
Before treatment
After treatment
T
T24-CIS+Vehicle
T24-CIS+Cisplatin
shNC
sh-circFOXK2#2
MTCO1

Figure S7. circFOXK2 promotes TACO1 mitochondrial translocation, stemness, and cisplatin resistance in BCa cells. (A) GST pull-down assays detected the interaction of TACO1 and HSP90β. **(B)** RIP-qPCR demonstrated the circRNAs that bind with HSP90β in T24-CIS cells. Data are shown as the mean ± SD, n = 3 (***p < 0.001, n.s. = non-significant, independent Student's t-test). **(C, D)** qRT-PCR revealed that after treatment with RNase R or Actinomycin D, the circFOXK2 and FOXK2 mRNA expression levels in T24-CIS cells. Data are shown as the mean ± SD, n = 3 (****p <0.0001, independent Student's t-test (C) and two-way ANOVA (D). **(E)** Cytoplasmic and nuclear fractionation experiments revealed the distribution of circFOXK2 in both the nucleus and the cytoplasm. β-actin and U3 were used as positive controls for the cytoplasm and nucleus, respectively. Data are shown as the mean ± SD, n = 3. **(F)** qRT-PCR verified the shRNAs knockdown efficiency of circFOXK2 and FOXK2 mRNA. Data are shown as the mean ± SD, n = 3 (****p <0.0001, n.s. = non-significant, independent Student's t-test). **(G)** co-IP revealed the interaction between TACO1 and HSP90AB1 after knockdown of circFOXK2 or treatment with RNase A. **(H)** Western blot analysis was conducted to assess the impact of circFOXK2 knockdown on TACO1 expression in both mitochondria and whole cells. COX IV was used as a mitochondrial marker, α-Tubulin was used as a cytoplasm marker and Histone-H3 was used as a nuclear marker. **(I)** IF showed the co-localization of TACO1 and mitotracker after knockdown of circFOXK2. Line graphs indicate the signal intensity of each protein along the arrow bar. Scale bar, 10 µm. **(J)** (Left) Western blot revealed differences in the expression levels of MTCO1 between shNC and sh-circFOXK2 groups in T24-CIS cells. (Right) Detection of the grayscale values of related proteins. Data are shown as the mean ± SD, n = 3 (***p < 0.001, independent Student's t-test). **(K)** Effect of circFOXK2 on mitochondrial complex IV activity. Data are shown as the mean ± SD, n = 3 (*p <0.05, independent Student's t-test). **(L)** Seahorse assays demonstrated alterations in OCR in T24-CIS cells after circFOXK2

knockdown. O: Oligomycin; F: FCCP; R/A: Rotenone & Antimycin A. Data are shown as the mean ± SD, n = 3 (***p <0.001, independent Student's t-test). **(M)** The flow cytometry experiment demonstrated the effect of circFOXK2 knockdown on the MitoSOX in T24-CIS cells. Data are shown as the mean ± SD, n = 3 (***p <0.001 independent Student's t-test). **(N)** The impact of circFOXK2 knockdown on the sphere-forming ability of T24-CIS cells. Data are shown as the mean ± SD, n = 3 (***p <0.001, independent Student's t-test). **(O, P)** The flow cytometry experiment demonstrated the effect of circFOXK2 knockdown on the proportion of ALDH1+ and CD44+ cell subpopulation in T24-CIS cells. Data are shown as the mean ± SD, n = 3 (**p <0.01, ***p <0.001 independent Student's t-test). **(Q)** The effect of circFOXK2 knockdown on cisplatin resistance in T24-CIS cells. Cell viability was assessed using a CCK-8 assay, and IC50 values were calculated through nonlinear regression analysis. Data are shown as the mean ± SD, n = 3**.** **(R)** (Left) Effects of knocking down circFOXK2 on the expression of stemness-related genes. (Right) Detection of the grayscale values of related proteins. Data are shown as the mean ± SD, n = 3 (**p < 0.01, independent Student's t-test). **(S, T)** Representative bioluminescence (Left), HE staining and IHC images (Right) and statistical results for the bioluminescence signals (Middle:) of the bladder orthotopic xenograft model were treated with vehicle or cisplatin in the corresponding subgroups. Scale bar, 100 μm. Data are shown as the mean ± SD, n = 5 (****p < 0.0001, n.s. = non-significant, independent Student's t-test).

Figure S8

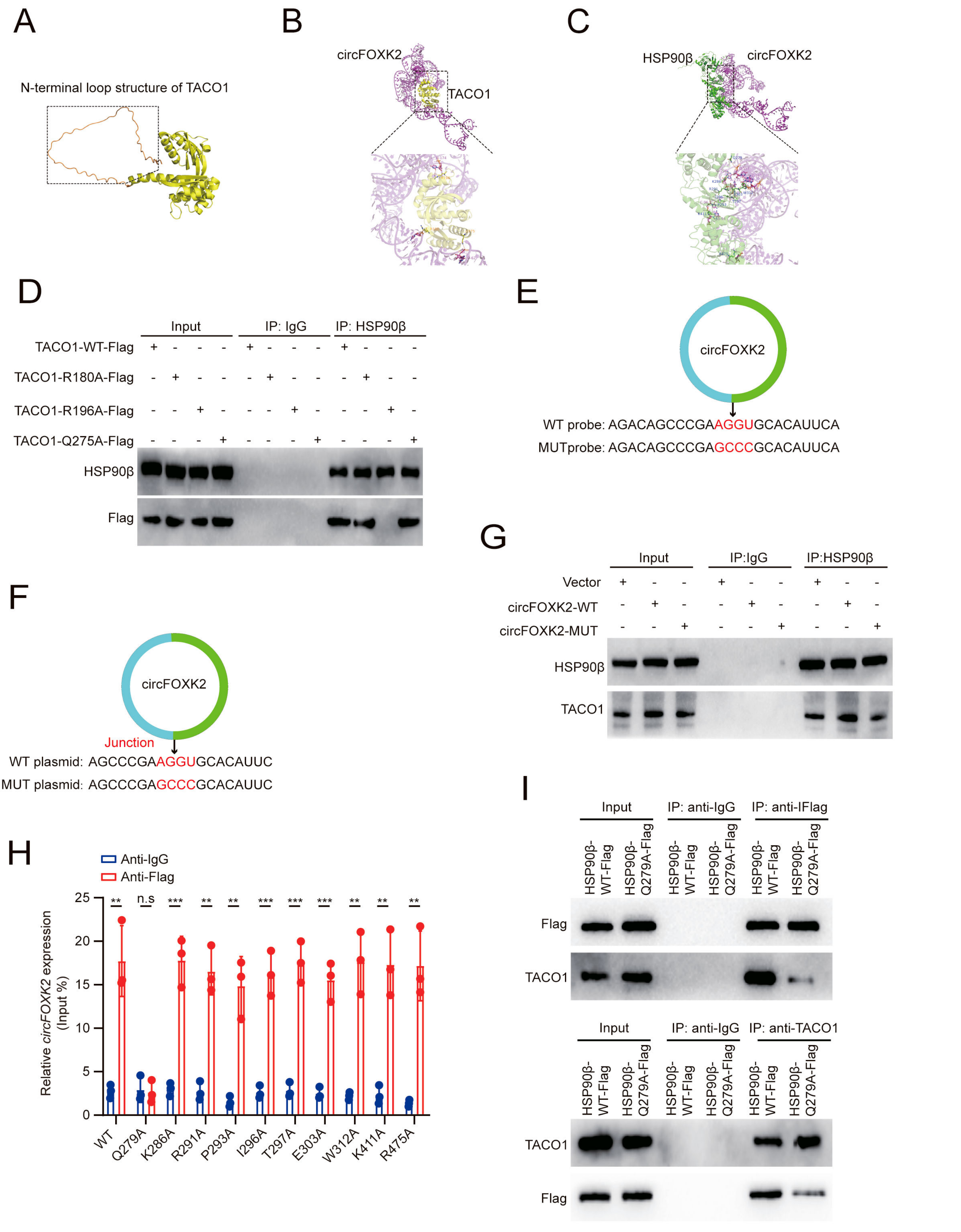
A
N-terminal loop structure of TACO1
B
circFOXK2
TACO1
C
HSP90β
circFOXK2
D
Input
IP: IgG
IP: HSP90β
TACO1-WT-Flag
TACO1-R180A-Flag
TACO1-R196A-Flag
TACO1-Q275A-Flag
HSP90β
Flag
E
circFOXK2
WT probe: AGACAGCCCGAAGGUGCACAUUCA
MUTprobe: AGACAGCCCGAGCCCGCACAUUCA
F
circFOXK2
Junction
WT plasmid: AGCCCGAAGGUGCACAUUC
MUT plasmid: AGCCCGAGCCCGCACAUUC
G
Input
IP:IgG
IP:HSP90β
Vector
circFOXK2-WT
circFOXK2-MUT
HSP90β
TACO1
H
Anti-IgG
Anti-Flag
Relative circFOXK2 expression (Input %)
** n.s *** ** ** *** *** *** ** ** **
WT Q279A K286A R291A P293A I296A T297A E303A W312A K411A R475A
I
Input
IP: anti-IgG
IP: anti-IFlag
HSP90β-WT-Flag
HSP90β-Q279A-Flag
Flag
TACO1
Input
IP: anti-IgG
IP: anti-TACO1
TACO1
Flag

Figure S8. HSP90β-circFOXK2-TACO1 binding site mutations disrupted the ternary complex. (A) The 3D structure of the full-length TACO1 sequence was predicted using AlphaFold2. The dotted box shows the loop structure at the N-terminal. **(B, C)** The molecular docking between TACO1 and circFOXK2, HSP90AB1 and circFOXK2 by HDOCK sever. **(D)** co-IP showed the ability of WT and MUT TACO1 to interact with HSP90β. **(E)** The schematic illustration showed the WT or MUT probe sequence of circFOXK2. **(F)** The schematic illustration showed the AGGU motif located at the exon 3−exon 2 junction site of circFOXK2 (Top). The junction site sequence of circFOXK2-WT and circFOXK2-MUT plasmid (Bottom). **(G)** co-IP showed the effects of WT or MUT circFOXK2 on the binding between TACO1 and HSP90β. **(H)** RIP-qPCR assay to evaluate the variations in binding affinity of wild-type HSP90β and each mutation towards circFOXK2. Data are shown as the mean ± SD, n = 5 (**$p < 0.01$, ***$p < 0.001$, n.s. = non-significant, independent Student's *t*-test). **(I)** The interaction between TACO1 and either the wild-type or mutant HSP90β was detected via co-IP.

Figure S9

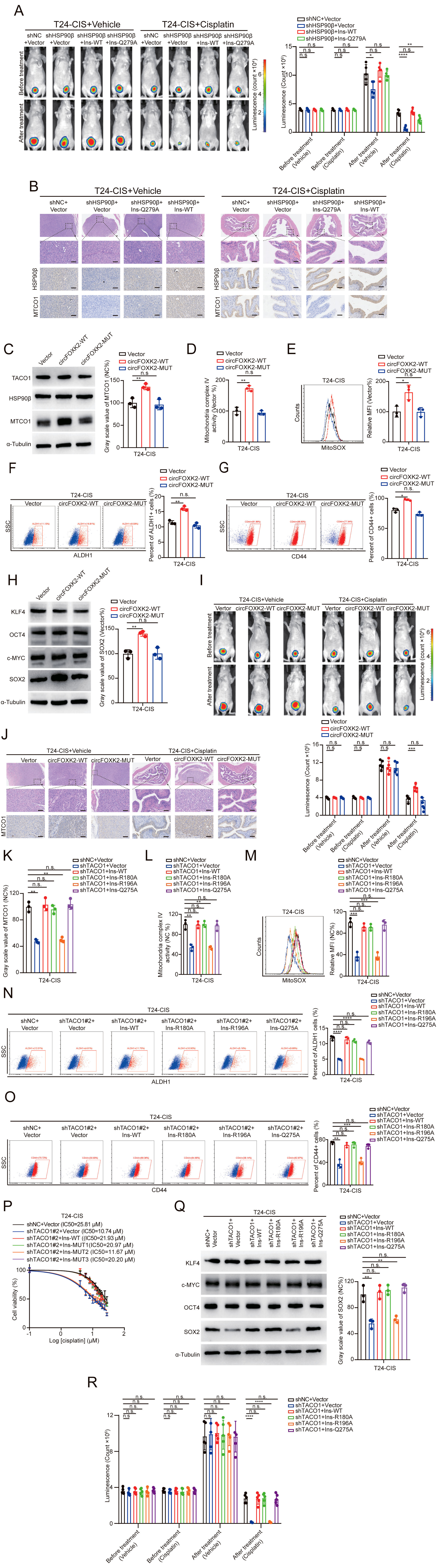

Figure S9. HSP90β-circFOXK2-TACO1 binding site mutations inhibit stemness and cisplatin resistance in BCa cells. (A) (Left) Representative bioluminescence and (Right) statistical results for the bioluminescence signals of the bladder orthotopic xenograft model were treated with vehicle or cisplatin in the corresponding subgroups. Data are shown as the mean ± SD, n = 5 (*$p < 0.05$, **$p < 0.01$, ****$p < 0.0001$, n.s. = non-significant, independent Student's *t*-test). **(B)** Representative HE staining and IHC images of the bladder orthotopic xenograft model. Scale bar, 100 μm. **(C)** (Left) Western blot showed the impact of overexpressing WT or MUT circFOXK2 on the expression of MTCO1 in T24 cells, (Right) Detection of the grayscale values of related proteins. Data are shown as the mean ± SD, n = 3 (**$p < 0.01$, n.s. = non-significant, independent Student's *t*-test). **(D)** Effect of circFOXK2-WT and circFOXK2-MUT on mitochondrial complex IV activity compared with Vector. Data are shown as the mean ± SD, n = 3 (**$p < 0.01$, n.s. = non-significant, independent Student's *t*-test). **(E–G)** The flow cytometry experiment showed the effect of WT or MUT circFOXK2 on the MitoSOX and the proportion of ALDH1+ or CD44+ cell subpopulation in T24-CIS cells. Data are shown as the mean ± SD, n = 3 (*p <0.05, **p <0.01, n.s. = non-significant, independent Student's t-test). **(H)** (Right) Effects of circFOXK2-WT and circFOXK2-MUT on the expression of stemness-related genes compared with Vector, (Left) Detection of the grayscale values of related proteins. Data are shown as the mean ± SD, n = 3 (**$p < 0.01$, n.s. = non-significant, independent Student's *t*-test). **(I)** Representative bioluminescence (Top) and statistical results for the bioluminescence signals (Bottom) of the bladder orthotopic xenograft models were treated with vehicle or cisplatin in the Vector, circFOXK2-WT and circFOXK2-MUT groups. Scale bar, 100 μm. Data are shown as the mean ± SD, n = 5 (***$p < 0.001$, n.s. = non-significant, independent Student's *t*-test). **(J)** Representative HE staining and IHC images (Right) of the bladder orthotopic xenograft models. **(K)** Detection of the grayscale values of related proteins. Data are shown as the mean

± SD, n = 3 (**p < 0.01, n.s. = non-significant, independent Student's t-test). **(L)** Effect of WT or MUT TACO1 on mitochondrial complex IV activity compared with shNC in T24-CIS cells. Data are shown as the mean ± SD, n = 3 (**p <0.01, n.s. = non-significant, independent Student's t-test). **(M–O)** The flow cytometry experiment showed the effect of WT or MUT TACO1 on the MitoSOX and the proportion of ALDH1+ or CD44+ cell subpopulation in T24-CIS cells. Data are shown as the mean ± SD, n = 3 (**p <0.01, ***p <0.001, ****p <0.0001, n.s. = non-significant, independent Student's t-test). **(P)** Corresponding cells were cultured with increasing concentrations of cisplatin for 48 hours. Cell viability was assessed using a CCK-8 assay, and IC50 values were calculated through nonlinear regression analysis. Data are shown as the mean ± SD, n = 3**. (Q)** (Left) Effect of WT or MUT TACO1 on stemness-related genes compared with shNC in T24-CIS cells. (Right) Detection of the grayscale values of related proteins. Data are shown as the mean ± SD, n = 3 (**p < 0.01, n.s. = non-significant, independent Student's t-test). **(R)** Statistical results for the bioluminescence signals. Data are shown as the mean ± SD, n = 5 (****p < 0.0001, n.s. = non-significant, independent Student's t-test).

Supplementary Tables

Table S1. Correlation expression of TACO1 and clinicopathological variables in 162 cases of BCa patients in SYSUCC.

Variable	All cases (N=162)	TACO1 expression (%)		P value [a]
		Low expression (%) (N=63)	Overexpression (%) (N=99)	
Age(years)				
≤ 60	86	37 (58.73%)	49 (49.49%)	0.251
>60	76	26 (41.27%)	50 (50.51)	
Gender				
Female	20	9 (14.29%)	11 (11.11%)	0.549
Male	142	54 (85.71%)	88 (88.89%)	
Smoking history				
No	79	30 (47.62%)	49 (49.49%)	0.816
Yes	83	33 (52.38%)	50 (50.51%)	
pT status				
T1-T2	84	45 (71.43%)	39 (39.39%)	0.000
T3-T4	78	18 (28.57%)	60 (60.61%)	
pN status				
pN-	122	54 (85.71%)	68 (68.69%)	0.014
pN+	40	9 (14.29%)	31 (31.31%)	
Histological Grade (WHO, 2004)				
Low grade	50	30 (47.62%)	20 (20.20%)	0.000
High grade	112	33 (52.38%)	79 (79.80%)	
Recurrence				
No	103	46 (73.02%)	57 (57.58%)	0.046
Yes	59	17 (26.98%)	42 (42.42%)	

[a] Chi-square test; BCa: bladder cancer.

Table S2. Univariate and multivariate analysis of different prognostic parameters in 162 patients with BCa in SYSUCC.

Variable	All cases	Univariate analysis[a]	P value	Multivariate analysis [b]	P value
		HR (95% CI)		HR (95% CI)	
Age(years)			0.014		0.016
≤60	86	1		1	
＞60	76	1.832 (1.108-3.029)		1.869 (1.124-3.106)	
Gender			0.194		
Female	20	1			
Male	142	1.812 (0.880-3.732)			
Smoking history			0.706		
No	79	1			
Yes	83	1.099 (0.671-1.801)			
pT status			0.001		0.007
T1-T2	84	1		1	
T3-T4	78	2.668 (1.589-4.481)		2.147 (1.231-3.745)	
pN status			0.006		0.721
pN-	122	1			
pN+	40	2.125 (1.039-4.347)			
Histological grade (WHO, 2004)			0.064		0.831
Low grade	50	1			
High grade	112	1.694 (1.015-2.829)			
TACO1			0.001		0.008
Low expression	63	1		1	
Overexpression	99	2.831 (1.723-4.650)		2.315 (1.242-4.313)	
Recurrence			0.001		0.001
No	103	1		1	
Yes	59	4.431 (2.580-7.611)		3.849 (2.270-6.526)	

[a] Univariate Cox regression model; [b] Multivariate Cox regression model; CI: confidence interval; HR: hazards ratio; BCa: bladder cancer.

Table S3. The contact list between circFOXK2, TACO1 and HSP90β

Chain 1	Residue	Chain 2	Residue	Interaction type
TACO1	Arg180: NH1	circFOXK2	C330: OP1	Hydrogen bond
TACO1	Arg196: NH1	circFOXK2	U174: O2	Hydrogen bond
TACO1	Gln275: NE2	circFOXK2	G62: OP2	Hydrogen bond
circFOXK2	G94: O4'	HSP90β	Lys411: NZ	Hydrogen bond
circFOXK2	C96: O2	HSP90β	Pro293: CD	Hydrogen bond
circFOXK2	U157: OP1	HSP90β	Gln279: NE2	Hydrogen bond
circFOXK2	A164: O2'	HSP90β	Trp312: NE1	Hydrogen bond
circFOXK2	C167: C4'	HSP90β	Glu303: OE1	Hydrogen bond
circFOXK2	A235: OP1	HSP90β	Arg291: NH2	Salt bridge
circFOXK2	A235: OP1	HSP90β	Ile296: CA	Hydrogen bond
circFOXK2	A235: OP1	HSP90β	Thr297: OG1	Hydrogen bond
circFOXK2	G236: O4'	HSP90β	Lys286: NZ	Hydrogen bond
circFOXK2	G236: OP1	HSP90β	Arg291: NH2	Salt bridge
circFOXK2	U322: OP1	HSP90β	Arg475: NH1	Hydrogen bond

Table S4: **The target sequences of si/shRNA used in our study.**

Gene name	Primer sequence (5′-3′)
ShTACO1#1	5′-GCACAACAAGTGGTCCAAAGT -3′
ShTACO1#2	5′-GAGGCATTATCTAACAGTA -3′
ShHSP90β#1	5′-GAAGGAACGAGAGAAGGAAAT-3′
ShHSP90β#2	5′-GGAACGAGAGAAGGAAATTAG -3′
ShCircFOXK2#1	5′-GACAGCCCGAAGGTGCACATT-3′
ShCircFOXK2#2	5′-AGACAGCCCGAAGGTGCACAT-3′

Table S5. **The primer sequences used to perform quantitative real-time PCR in our study.**

Primer Name	Direction	Primer sequence (5'-3')
MT-CO1	Forward	CAGCAGTCCTACTTCTCCTATCTCT
	Reverse	GGGTCGAAGAAGGTGGTGTT
β-actin	Forward	GAGAAAATCTGGCACCACACC
	Reverse	GGATAGCACAGCCTGGATAGCAA
U3	Forward	TTCTCTGAGCGTGTAGAGCACCGA
	Reverse	GATCATCAATGGCTGACGGCAGTT
GAPDH	Forward	GTCAGTGGTGGACCTGACCT
	Reverse	TGCTGTAGCCAAATTCGTTG
circCLIP2	Forward	ACAAGCCACATCTGCGAGG
	Reverse	AGCCGATACGGATCACTTTGT
circFOXK2	Forward	ATCAGCGCTGCAAACTCCTG
	Reverse	TGGACAGGGCAGTGAACGTT
circXPO1	Forward	AGATTCTTCCAAGGAACCAGTGC
	Reverse	CTGGGCTCCTTCTCCATGGTAT
circANKRD12	Forward	AACAGAGGAGTTGGAGTTGCT
	Reverse	TCCTTGCTCACATCACTTCGT
circABR	Forward	AGTTTGAGATCGAGCTGGAGGG
	Reverse	TTCCTCATCTCCAGGCCTTTCC
circMAN1A2	Forward	GGGGACATAATGAACTCAGACCT
	Reverse	GCTTCTTCCAAGGCCTTCTCA
circMYO9B	Forward	CGTGAACCAGTGCATCGTGATC
	Reverse	TGCCTGCCTCTTTCACACTCAT
circANKRD11	Forward	AAGACCCCAAAACTGGAGCG
	Reverse	TCTTCATTTACACGGCCGGC
circSENP6	Forward	GCAGGAAGAAAGTGACCCTCGT
	Reverse	ACCGGGGAAACAAACAACAGC
circKMT2C	Forward	GTGGTCACTAGGAGTATGCCAGA
	Reverse	AGGTTCCATTGCTGTTGTCATCA
FOXK2	Forward	CAGGAGTTTGCAGCGCTGAT
	Reverse	AACGTTCACTGCCCTGTCCA
